# Vinculin influences essential processes in enteric nervous system development and Hirschsprung disease pathogenesis

**DOI:** 10.1172/JCI198531

**Published:** 2025-12-09

**Authors:** Lifang Liu, Xixin Wang, Mingxuan Liang, Peiting Li, Cindy Yifei Yan, Patrick Ho-Yu Chung, Kenneth Kak-Yuen Wong, Asif Javed, Maria-Mercedes Garcia-Barcelo, Elly Sau-Wai Ngan

**Affiliations:** 1 Department of Surgery and; 2 School of Biomedical Science, Li Ka Shing Faculty of Medicine, The University of Hong Kong, Pokfulam, Hong Kong, China.; 3 Translational Research Unit, Chulabhorn Research Institute, Bangkok, Thailand.

**Keywords:** Gastroenterology, Genetics, Neurodevelopment

## Abstract

Vinculin (VCL), a linker between cells and their environment, has rarely been linked to disease. This study examines the role of VCL in the development of the enteric nervous system (ENS) and its relationship to Hirschsprung disease (HSCR). Using whole-genome sequencing and in vitro assays, we identified 4 *VCL* mutations associated with HSCR, most causing loss of function. Neural crest–specific *Vcl* knock-out mice (*Vcl* cKO) displayed ENS defects resembling short-segment HSCR, including partial colonic aganglionosis and abnormal gut musculature. Single-cell transcriptomics revealed dysregulation of genes involved in neuronal differentiation and MAPK signaling. Spatial RNA-seq revealed reduced ENS-mesenchyme interactions in *Vcl* cKO mice, accompanied by significant disruption of the Pleiotrophin (PTN) pathway; *Ptn* knock-out mice exhibited phenotypes similar to those of *Vcl* cKO mice, underscoring the importance of ENS-mesenchyme crosstalk. VCL works as a hub gene crucial for cell connection and signaling pathways essential for ENS formation. *VCL* deficiency subtly impacts various developmental stages and neighboring cells, cumulatively leading to a phenotype similar to short-segment HSCR. This research highlights the role of VCL in maintaining cellular interactions and signaling pathways, such as MAPK and PTN, which are crucial for ENS development and may inform therapeutic targets for ENS disorders.

## Abstract 2

Vcl deficiency influences multiple pathways in ENS formation, subtly affecting development and nearby cells. These small effects lead to a phenotype with partial aganglionic bowel, as seen in S-HSCR.

## Graphical abstract

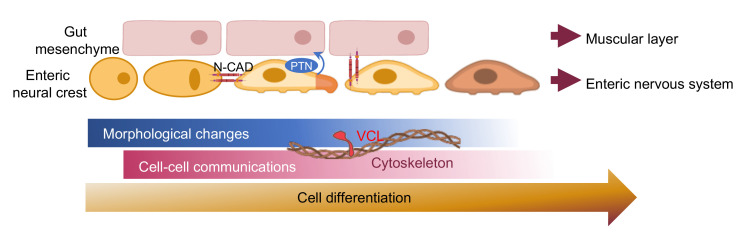

## Introduction

Hirschsprung disease (HSCR) is the most common neurocristopathy, affecting approximately 1 to 1.3 out of every 5,000 newborns (1). This complex congenital disorder impacts the colon, resulting in the absence of nerve cells along a variable length of the colon due to incomplete colonization by enteric neural crest cells (ENCCs), which ultimately leads to functional intestinal blockage. Patients with a short aganglionic segment (S-HSCR), which includes the distal rectum and extends up to the rectosigmoid region, represent the most common form of HSCR, making up approximately 80% of cases. Long segment (L-HSCR) and total colonic aganglionosis (TCA) are the more severe forms, representing the remaining 20% of the cases.

Neural crest cells (NCCs) derived from the vagal region of the neural tube constitute the primary population of progenitor cells for the enteric nervous system (ENS) and begin to enter the esophagus around embryonic day 9.5 to 10.5 in mice. These cells proliferate extensively, migrate distally over long distances to colonize the developing gut, and differentiate into millions of neurons and glia, which are organized into a network to control bowel functions (2). A precise coordination of cell differentiation, migration, and ganglionogenesis is essential for the proper formation of a functional ENS (3).

Cell-to-cell communication signals among various cell populations are crucial for the development of the ENS. However, there remains limited knowledge about the specific molecules and signals involved in the interactions between different ENS cells and their neighboring cells that guide ENS development. To date, only a few homophilic adhesion molecules, such as N-cadherin, NCAM, and the L1 cell adhesion molecule (L1CAM), have been linked to ENCC migration. The loss of either N-cadherin or L1CAM leads to delayed ENCC migration and malformation of enteric aganglionosis (4). Moreover, the posttranslational modification of NCAM with polysialic acid (psNCAM) affects the aggregation and migration efficiency of ENCCs (5, 6), but neither factor alone is sufficient to cause aganglionosis.

Vinculin (VCL) was identified as a crucial component of focal adhesions (FAs) and adherents junctions (AJs), playing a key role in mediating cell-matrix and cell-cell adhesions, respectively (7). It exists in 2 isoforms: the shorter VCL isoform, which is widely expressed, and the longer, muscle-specific metavinculin (meta-VCL). VCL interacts with over 14 putative binding partners at FAs, including talin, actin, and paxillin, to facilitate various cellular functions (8, 9). These interactions are essential for positioning VCL at the integrin signaling level, where it can respond to external stimuli (10). Moreover, these interactions are regulated by an autoinhibitory mechanism involving intramolecular interactions between the head and tail domains of VCL (11, 12). The release of this autoinhibition is necessary for the simultaneous binding of multiple ligands (13). On the other hand, VCL mediates cell-cell adhesion through a multistep process involving α-catenin, β-catenin, and YAP1 (7, 14). *VCL*-null mutants exhibit embryonic lethality by day 10 due to failures in rostral neural tube closure, abnormalities in forelimb development, heart malformations with reduced size, and fewer myocytes than normal (15), and compromised development of cranial and spinal nerves (16).

A potential causal link between VCL and HSCR was first illustrated in our previous study, in which a mutation (M209L) in the *VCL* gene was identified in a syndromic patient presenting with HSCR disease and congenital cardiac defects, including ventricular septal defects (VSD) and valvular abnormalities (17). Subsequent functional analyses utilizing an induced pluripotent stem cell–based (iPSC-based) model demonstrated that the M209L substitution disrupts focal adhesion (FA) assembly, resulting in impaired migration and deficient neuronal/smooth muscle lineage differentiation of patient-specific iPSC-derived neural crest cells (NCCs). Correcting the *VCL* mutation effectively rescued the FA phenotype, leading to the restoration of NCC functions (17). At the molecular level, the M209L substitution disrupted the VCL-ACTIN interaction, likely interfering with the morphological changes of the cells associated with various developmental processes (17). However, it remains largely unclear whether the ablation of *VCL* alone is sufficient to cause disease and its potential implications for the pathogenesis of HSCR.

In this study, we first reanalyzed the exome regions of the genome using our in-house whole-genome sequencing dataset of S-HSCR (18, 19) to explore the relevance of *VCL* in the pathogenesis of HSCR. Subsequently, we generated a mouse model with NCC-specific ablation of *VCL* to directly demonstrate its implications in ENS development. High-resolution and spatial RNA-seq data further elucidated the molecular mechanisms involved and the sequential effects resulting from the loss of cell-cell and cell-matrix interactions. This highlighted the significance of interactions among ENS cells and their surrounding environment in both ENS formation and HSCR pathogenesis. Intriguingly, VCL does not operate through a single, dominating effect via a specific signaling pathway, as is often the case in Mendelian diseases. Instead, it functions as a hub gene, integrating various cellular processes during development, which can subsequently lead to disease when disrupted.

## Result

### Multiple loss-of-function mutations in VCL were found in patients with both syndromic and isolated HSCR.

**Figure 1:**
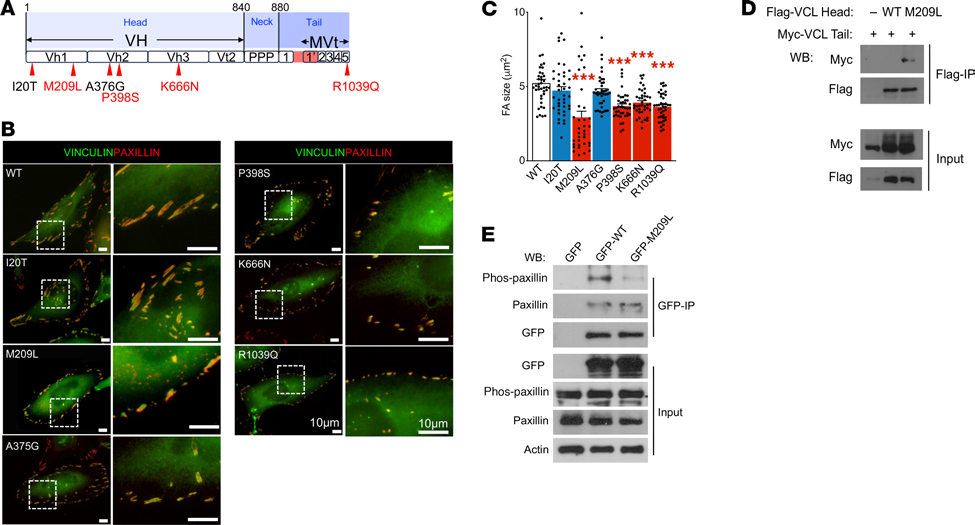
HSCR-associated mutations in *VCL* interrupt focal adhesion assembly. (**A**) The schematic shows that mutations affect different functional domains of the VCL protein. (**B**) Immunofluorescent stains show the focal adhesions (FAs) as marked by colocalization of Paxillin and Vinculin in HeLa cells overexpressing various *VCL* mutants. Scale bars: 10 μm. (**C**) Bar graph shows the average FA size found in cells overexpressing various *VCL* mutants (mean ± SEM, > 50 cells were analyzed; ***: *P* < 0.001, 1-way ANOVA). co-IP assay indicates that M209L substitution in VCL (**D**) enhances head and tail interaction, (**E**) leading to reduced binding affinity to phosphorylated paxillin.

To elucidate the role of *VCL* in the pathogenesis of Hirschsprung disease (HSCR), we reanalyzed a whole-genome sequencing dataset comprising 94 patients with S-HSCR with comprehensive clinical records, which included 13 patients presenting with both HSCR and VSD, representing approximately 13% of the cohort (19). In total, we identified 7 de novo heterozygous mutations distributed across various exons of the *VCL* gene in 9 patients with HSCR (Figure 1A and Supplemental Data 1; supplemental material available online with this article; https://doi.org/10.1172/JCI198531DS1). Among these, A977P mutation is located within the functional domain of the muscle-specific isoform (isoform 1), which is not expressed in NCCs (Supplemental Figure 1), it is plausible that this mutation does not contribute to NCC-associated defects.

We then evaluated the potential impacts of the remaining 6 *VCL* mutations on focal adhesion (FA) assembly by measuring FA sizes, as described in previous studies (17). In brief, expression constructs carrying either WT or mutant *VCL* were overexpressed in the human cervical cancer cell line HeLa, and FAs were detected using immunocytochemistry with the FA marker Paxillin. The sizes of the FAs were measured and compared. Notably, 4 out of 6 mutations were found to be deleterious, leading to a reduction in FA size, primarily located within the head or tail domains of VCL (Figure 1, B and C, and Table 1). The M209L substitution was identified in 3 nonconsanguineous syndromic patients with HSCR presenting with VSD and exhibited a prominent effect on FA assembly. Therefore, we further investigated the mechanism by which the M209L substitution disrupts FA assembly. Specifically, this mutation is situated in head domain 1 (Vh1) of VCL, and it may promote autoinhibitory head-to-tail interactions. To test this hypothesis, we generated expression constructs incorporating either the head (amino acids 1–258) or tail (amino acids 879–1,066) regions of VCL, tagged with FLAG or c-MYC, respectively. Both WT and M209L mutant head constructs, along with the c-MYC–tagged tail construct, were coexpressed in HeLa cells, followed by coimmunoprecipitation assays. Our findings revealed that the M209L substitution enhances the head-to-tail interaction, likely resulting in VCL inactivation (Figure 1D). Moreover, VCL mutants exhibited a diminished binding affinity to phosphor-Paxillin (Figure 1E), which may account for the observed FAs defects. Importantly, impaired FA assembly may reduce the ability of cells to respond to external stimuli and interact with their environment, thereby disrupting various cellular processes.

### Neural crest–specific ablation of VCL caused partial colonic aganglionosis.

**Figure 2:**
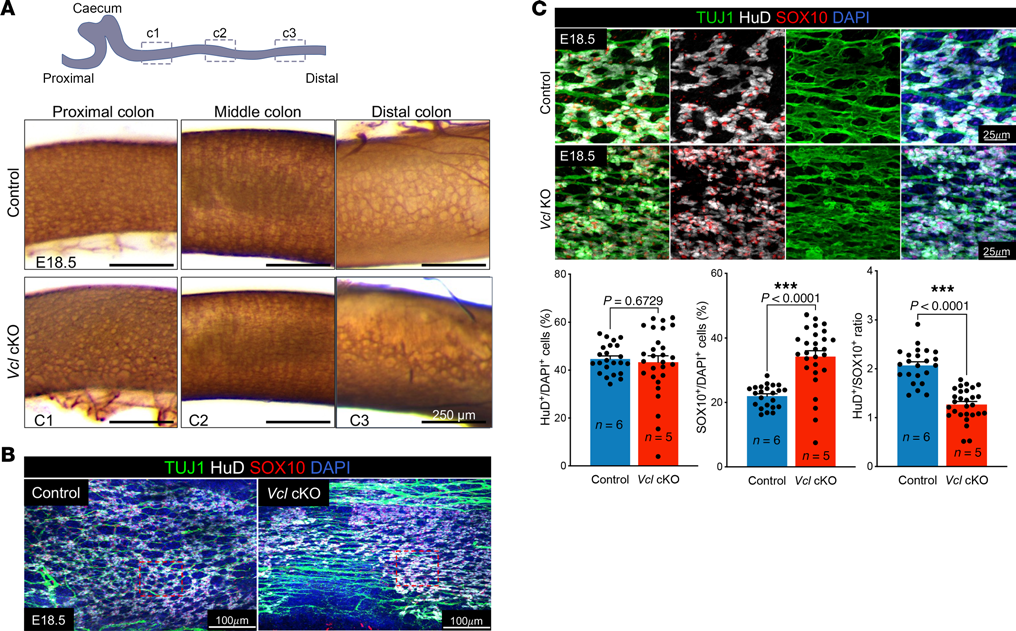
Malformation of enteric ganglia in *Vcl* cKO at E18.5. (**A**) Three regions (proximal: C1; middle: C2 and distal: C3) of colon were collected from control and *Vcl* mutants (*Vcl* KO*^Wnt1–Cre^*) for whole-mount acetylcholinesterase (AchE) staining. Scale bars: 250 μm. (**B**) Whole-mount immunofluorescence of TUJ1, HuD and SOX10 shows ENS network in the distal colon of E18.5 *Vcl* cKO was aberrantly organized. Scale bars: 100 μm.(**C**) Magnified images of the regions highlighted in the red dotted boxes in **B**. Scale bars: 25 μm. The percentages of neurons (HuD^+^) and glia (SOX10^+^) normalized to the total number of cells (DAPI^+^) and the relative neuron-to-glia ratio in each region are shown in the bar charts. (mean ± SEM, n: number of embryonic guts analyzed, ***: *P* < 0.001, Student *t* test, 2-tailed). 6–8 randomly selected regions were analyzed.

To directly investigate the roles of VCL in the development of ENCCs, both copies of the *Vcl* gene were specifically deleted from NCCs using the *Wnt1-Cre* mouse line, resulting in *Vcl* conditional knock-out (cKO) mutants. These mutants exhibited both cardiac and enteric nervous system (ENS) defects and did not survive beyond a few hours after birth. In terms of ENS phenotypes, nearly all E18.5 mutants analyzed (*n* > 20) showed varying degrees of colonic aganglionosis, as determined by acetylcholinesterase (AChE) staining. As demonstrated in Figure 2A, the ENS network did not fully cover the distal colon in the *Vcl* cKO mutants, whereas the ENS in the proximal and middle colon appeared unaffected and closely resembled that of the control group. We subsequently performed IHC analyses on whole-mount colon preparations, consistently revealing aganglionic regions in the distal colon of the *Vcl* cKO mutants (Figure 2B). Interestingly, the neurons and glial cells adjacent to these aganglionic areas were not properly organized nor distributed as ganglia, in contrast to the arrangement observed in the control (Figure 2C). We then quantified the number of HuD^+^ neurons and SOX10^+^ glial cells in the distal colon. In the control colon, both neurons and glial cells were evenly distributed with comparable density across 4–8 random views (600 mm² each) from 3 embryonic guts. The counts of neurons and glia were normalized against the total number of cells per view, as indicated by DAPI staining. In summary, the control group exhibited 41.84% ± 1.39% neurons and 22.65% ± 1.07% glia, resulting in a neuron-to-glia ratio of 1.89 ± 0.09. In contrast, in the comparable regions, the mutants displayed a lower percentage of neurons (34.56% ± 3.80%) and a higher percentage of glia (29.29% ± 3.07%), along with greater variability. The neuron-to-glia ratio in the mutants was significantly reduced to 1.20 ± 0.12 (Figure 2C).

**Figure 3:**
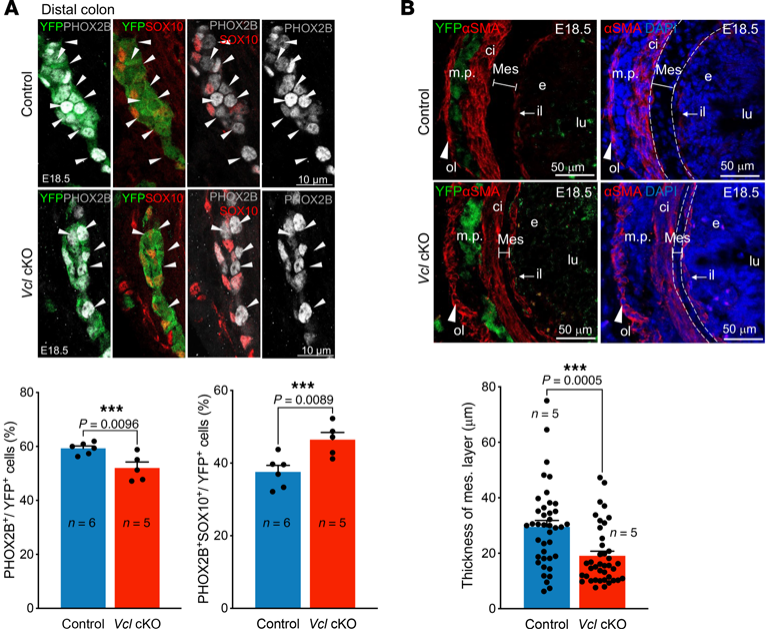
Immature ENS in *Vcl* cKO at E18.5. Immunofluorescence of (**A**) PHOX2B & SOX10; and (**B**) SMA and YFP counterstained with DAPI on cross sections of E18.5 distal colon, and the quantitative data are shown in bar graphs. (mean ± SEM; n, number of embryonic guts analyzed; *P* < 0.05 was considered significantly different; ***P < 0.001, Student *t* test, 2-tailed). 6–8 randomly selected regions were analyzed. Mes, mesenchyme; e, endoderm; lu, lumen; ci, circumferential muscle; ol, outer longitudinal layer; il, inner longitudinal layer; m.p., myenteric plexus. Scale bars: 10 μm (**A**); 50 μm (**B**).

We then generated *Vcl* cKO with a YFP background (*Wnt1-Cre; Rosa26^YFP^;Vcl^f/f^*), allowing for the labeling of ENCCs with YFP. Additional IHC analyses were performed on sections of the distal colon from E18.5 control and mutant specimens. Consistent with the data obtained from whole-mount staining, we observed a significant reduction in the percentage of committed neurons (PHOX2B^+^SOX10^–^YFP^+^), accompanied by an increase in the number of uncommitted ENCCs (PHOX2B^+^SOX10^+^YFP^+^) (Figure 3A). In sum, *Vcl* mutants displayed partial aganglionosis and exhibited an immature ENS.

Upon analyzing the crosssections of the mutant guts at E18.5, we observed that the smooth muscle in the *Vcl* cKO colon was less densely packed and less organized, with a notable reduction in the thickness of the mesenchymal layer (Figure 3B). Since *Vcl* was specifically deleted in NCCs, this is likely a secondary consequence of the ENS defect, suggesting that the ENS plays a crucial role in the development and patterning of the mesenchyme and smooth muscle.

### Aberrant migration of ENCCs in Vcl mutants.

**Figure 4:**
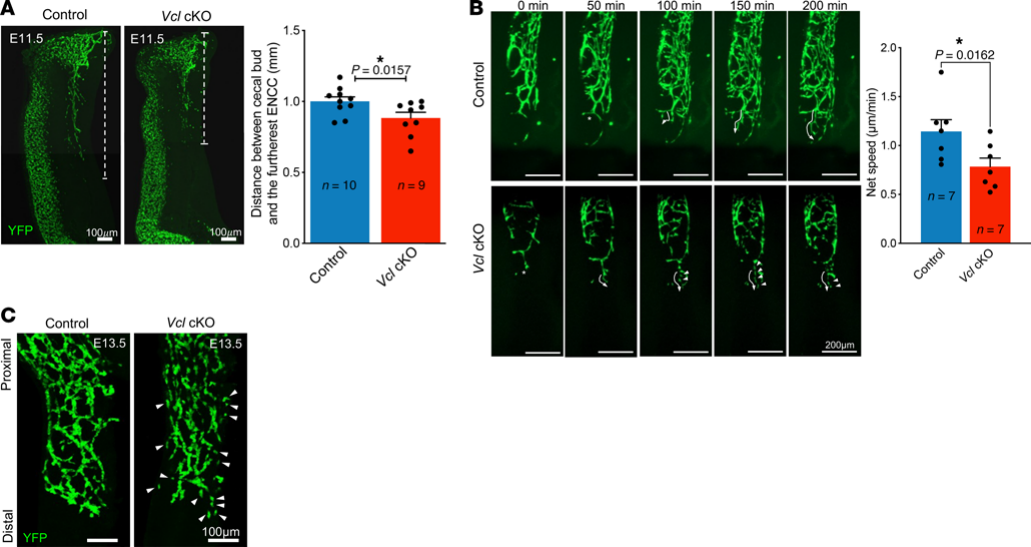
Failure to form the migration chain in *Vcl* mutants. (**A**) The migration of ENCCs in hindgut and colonization in cecum were examined at E11.5. White dashed lines indicate the migration distance. Bar graph shows significantly delayed migration of ENCCs in the mutant mice. *P* < 0.05 was considered significantly different and marked by *, Student *t* test, 2-tailed. Scale bar: 100 μm. (**B**) Time-lapse images from live imaging of ENCC migration in E12.5 embryonic guts, where YFP-labelled ENCCs located at the migratory wavefront (asterisks) were tracked and their migratory paths were indicated by arrows. Bar graph shows the net speed of migration. Scale bar: 200 μm. (**C**) Whole mount immunofluorescence of YFP-labeled ENCCs in E13.5 hindguts. Failure in formation of migratory chains and presence of solitary ENCCs (arrowheads) were observed in *Vcl* cKO. *P* < 0.5 was considered significantly different and marked by “*”, Student *t* test, 2-tailed. Scale bar: 100 μm.

We reasoned that the incomplete colonization of the distal colon in mutants would be the result of migration defects of ENCCs. Thus, we collected embryonic guts from E11.5 to E13.5, which is the key developmental window for gut colonization, and analyzed the migration of ENCCs in the ex vivo guts during these stages. A slight but significant delayed migration of ENCCs was observed in E11.5 *Vcl* cKO embryonic gut (Figure 4A).

To investigate their migration patterns, live imaging was conducted over a period of 12 to 15 hours using E12.5 hindguts to observe the behavior of YFP-labeled ENCCs in both control and mutant conditions. The control ENCCs exhibited a distinctive migration pattern, moving in chains that often converged and diverged as they elongated distally to invade the uncolonized regions of the gut. The nodes formed at the intersections of these ENCC chains, along with the interconnecting chains themselves, are proposed to play a role in guiding the arrangement of ganglia into a lattice-like neuronal network (20). ENCCs in the control gut remained interconnected as continuous strands throughout their migration (Figure 4B). In contrast, the migration of ENCCs was disrupted in *Vcl* cKO mutants. Although these mutant cells could migrate toward the distal end of the hindgut, they displayed an inconsistent migration trajectory. More significantly, the mutant cells failed to establish a migratory chain at the migratory front, resulting in a number of solitary ENCCs (Figure 4B and Supplemental Video 1). We then further monitored the behavior of ENCCs at the migratory front. While the migration direction of ENCCs in both *Vcl* cKO and control groups was similar, with a tendency to move toward the distal end of the hindgut, the mutant ENCCs consistently deviated from the primary migratory path (Supplemental Figure 2A). Additional analysis of their migration tracks revealed that the average speed of cell migration was somewhat reduced in the mutants (Supplemental Figure 2B), whereas their persistence (defined as the ratio of net distance traveled to total distance traveled) remained comparable with that of control cells (Supplemental Figure 2C). Alongside the irregular migratory patterns observed in VCL-deficient ENCCs, the overall net migration speed was also diminished (Figure 4B). By E13.5, a distinct neuronal meshwork had formed in the control gut; however, the mutant ENCCs were unable to maintain connections with neighboring cells, leading to a significant presence of solitary ENCCs at the distal end of the hindgut (Figure 4C). This observation indicates that the connections between ENCCs were disrupted during migration, which likely interferes with subsequent ganglionogenesis.

### Reconstruction of the differentiation paths of ENCCs reveals that loss of VCL delays their differentiation along the Branch A.

**Figure 5:**
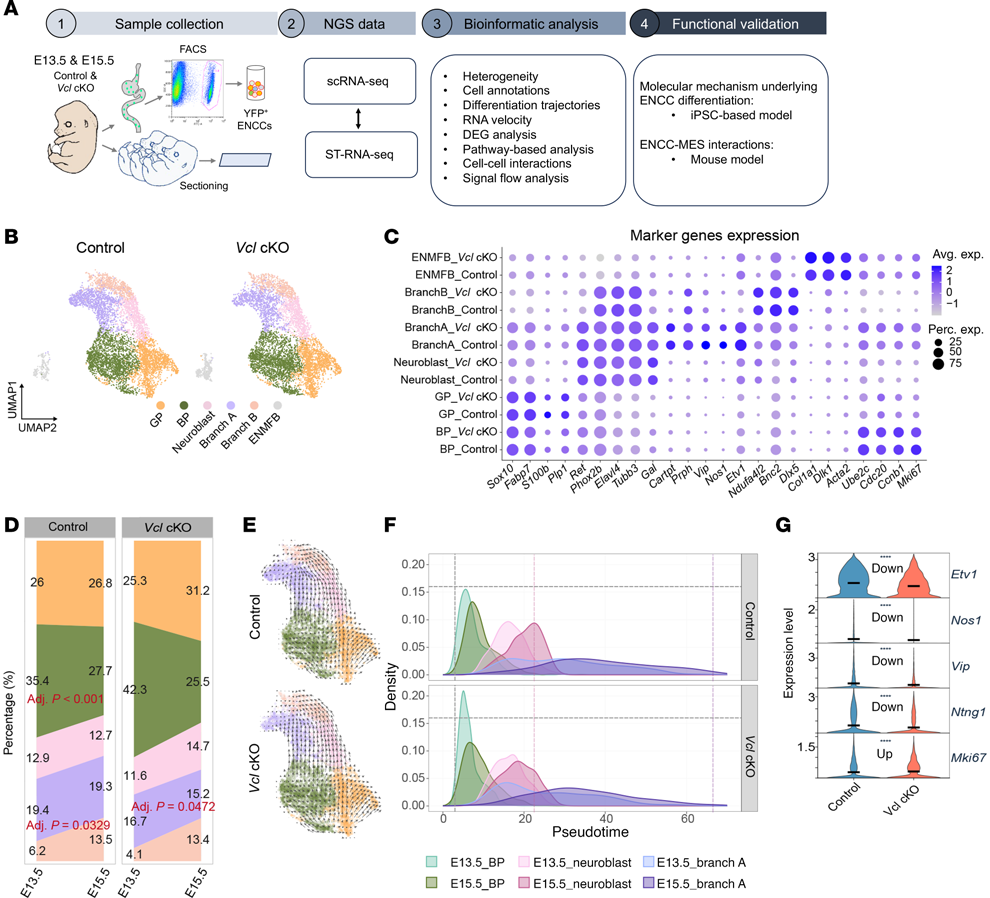
scRNA-seq analysis revealed a delayed progression of *Vcl* mutant cells along the neuronal lineage differentiation trajectory. (**A**) The workflow summarizing scRNA-seq data analysis and corresponding functional validations. (**B**) The integrated UMAP (Uniform Manifold Approximation and Projection) projection of 30,157 cells ENCCs at E13.5 and E15.5, colored by cell types. (BP, biopotent progenitor; GP, glial progenitor; Branch A & Branch B neurons, annotated by integrating the data from Morarach et al (46); ENMFB, enteric mesothelial fibroblast.) (**C**) The expression of marker genes and proliferative markers across cell types. (**D**) The proportion of cell types across different samples. Empirical Bayes moderated *t* test is used for the cell proportion comparison, with Benjamini-Hochberg–adjusted *P* value < 0.05 considered significant. The proportion of E13.5 BP, E13.5 Branch A, and E15.5 Branch A neurons is affected. (**E**) Comparison of RNA velocity in control and *Vcl* mutant. (**F**) The density plot shows the distributions of pseudotime of cell states inferred by Slingshot, revealing a delayed differentiation of neurons. (**G**) Expression of Branch A markers in control and *Vcl* mutant. 2-sided Wilcoxon rank-sum test, ****P < 0.0001.

In addition to the migration defect, the mutant exhibited a reduced number of mature neurons and an increased presence of uncommitted ENCCs, as illustrated in Figure 2C and Figure 3A. This suggests a potential differentiation defect in the mutant ENCCs. Notably, delayed differentiation was observed as early as E13.5, with the differentiation defect becoming more pronounced by E15.5 (Supplemental Figure 3). Therefore, we analyzed the transcriptomes of ENCCs that were isolated and enriched through fluorescence-activated cell sorting (FACS) from 7 guts of control (*Wnt1-Cre; Rosa26^YFP^*) and *Vcl* mutant (*Wnt1-Cre; Rosa26^YFP^;Vcl^f/f^*) embryos at E13.5 and E15.5 (Supplemental Figure 4), each from 2–3 litters. This was performed using single-cell RNA-seq with 10X Genomics to explore further how the loss of *Vcl* influences the molecular dynamics of ENCCs along their differentiation trajectories. Additionally, we carried out 10X Visium spatial transcriptomics (ST) analysis on sagittal sections of E13.5 embryos to investigate how alterations in signaling among various ENS cells and their neighboring cells contribute to the observed defects in the mutants. By cross referencing our scRNA-seq and ST-RNA-seq datasets, we aimed to clarify the molecular mechanisms by which VCL governs ENCC development and their interactions with adjacent cells. Human induced pluripotent stem cell (hiPSC) ENS-based and mouse models were then used for functional validation. A schematic summarizing our analysis pipeline is presented in Figure 5A.

After performing quality control on the scRNA-seq data, we identified a total of 30,157 cells, with each cell exhibiting an average of 3,677 genes and 13,860 unique molecular identifiers (UMIs) (refer to Supplemental Figure 5 and Supplemental Data 2). To refine the differentiation trajectories, we integrated our dataset with a previously published dataset of E18.5 ENS cells 21 and reannotated them based on the expression of canonical marker genes and lineage-specific transcriptional factors identified in the original study (Supplemental Figure 6). The Uniform Manifold Approximation and Projection (UMAP) plots revealed 5 cell clusters comprising 3 distinct differentiation branches: neuronal, which includes inhibitory (Branch A) and excitatory (Branch B) neurons, and glial lineages (Figure 5B). All paths originated from the highly proliferative bipotent progenitors (BP) characterized by high expression of *Mki67*, *Ube2c*, *Cdc20*, and *Ccnb1*. The cells on the neuronal differentiation trajectory progressed through a neuronal intermediate stage, Neuroblast, which is marked by early neuronal markers (*Tubb3^high^/Elavl4^high^/Cartpt^−^/Prph^−^*), before diversifying into 2 branches distinguished by the complementary expression of *Etv1* and *Bnc2*, representing Branch A and Branch B neurons, respectively. By E15.5, Branch A constituted the predominant neuronal population, coexpressing markers of inhibitory neurons like *Nos1* and *Vip*, while a smaller subset expressed *Bnc2* with a reduction in *Etv1* expression, corresponding to Branch B excitatory neurons. In terms of the glial lineage differentiation trajectory, glial progenitors (GPs) displaying high levels of *Sox10* and *Fabp7* began to emerge at this stage. Additionally, a distinct population of progenitors that expressed unique marker sets referring to the enteric mesothelial fibroblasts (ENMFBs) was identified (Figure 5, B and C, and Supplemental Data 3).

We subsequently examined the impact of *Vcl* loss on cell composition. Both the control and mutant groups contained all 5 clusters of neural cells and the ENMFB cluster. Interestingly, the *Vcl* mutant showed an increased proportion of BP cells at E13.5, coupled with a decrease in Branch A neurons at both E13.5 and E15.5 (Figure 5D). Further RNA velocity analyses revealed that the differentiation trajectories in both control and *Vcl* mutant populations were similar (Figure 5E), indicating that the absence of *Vcl* does not disrupt the differentiation of ENCCs toward a specific lineage, nor does it introduce a differentiation bias that would lead to a reduction in Branch A.

To investigate the heterogeneity of cells within the BP-to-Branch A lineage, we aimed to order the cells in pseudotime and infer the trajectories along this differentiation path. We began by performing a Principal Component Analysis (PCA) to reorganize all the cells in the BP-to-Branch A lineage. The distribution of cells projected to principal components 1 (PC1) and 3 (PC3) illustrated a continuous pseudotemporal trajectory from the BP to Branch A. Consequently, we inferred the pseudotime based on these 2 dimensions (Supplemental Figure 7, A–C). As shown in Figure 5F, both control and mutant cells from E13.5 and E15.5 exhibited a well-ordered arrangement along the pseudotime of differentiation. In the *Vcl* mutant, the BP cells at E13.5, Neuroblasts at E15.5, and Branch A neurons demonstrated significantly lower pseudotime values compared with the control cells, indicating a delayed differentiation along the Branch A neuronal lineage and immaturity of the Branch A neurons (Figure 5F and Supplemental Figure 7D). Consistently, a significant reduction in the expression of Branch A marker genes, coupled with elevated expression of the proliferative marker *Mki67*, was observed, suggesting fewer mature Branch A neurons in *Vcl* mutant (Figure 5G).

**Figure 6:**
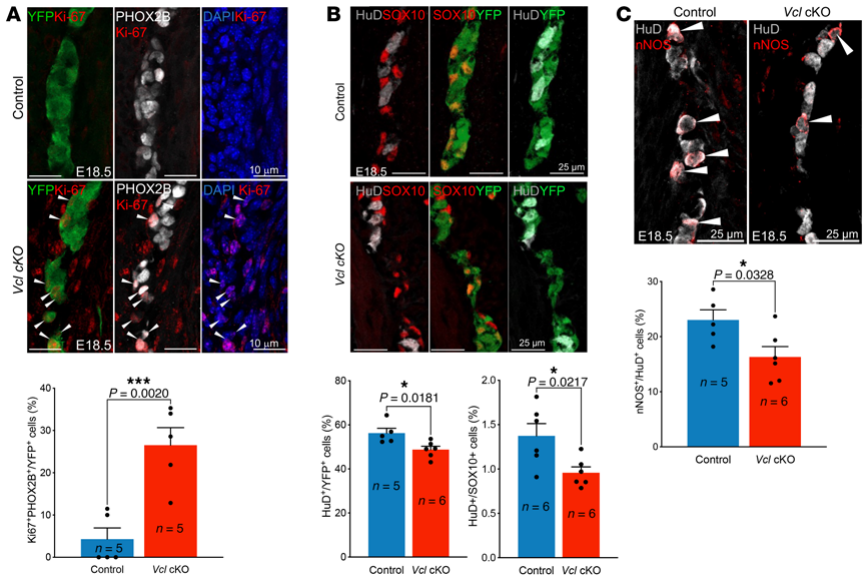
Increased progenitor cells and delayed differentiation in *Vcl* mutants. (**A**) Mitotic ENS neurons (Ki67^+^PHOX2B^+^YFP^+^); (**B**) mature (HuD^+^SOX10^–^) and immature (HuD^+^SOX10^+^) neurons; and (**C**) inhibitory (nNOS^+^) neurons. Bar graphs show the quantitative data (mean ± SEM; n, number of embryonic guts analyzed; *P < 0.05; ***P < 0.001, Student *t* test, 2-tailed; scale bars: 10 μm (**A**); 25 μm (**B**, **C**)).

In accordance with the RNA-seq data, a significantly greater number of ENCCs remained proliferative (Ki67^+^) in the mutants at E18.5, while only postmitotic ENCC derivatives were observed in the control group at this stage (Figure 6A). This finding further suggests the immature nature of the ENS in the mutants. Consistent with this observation, there were significantly fewer mature neurons (HuD^+^) and a larger population of immature neurons (HuD^+^SOX10^+^) in the mutants compared with the controls (Figure 6B). Notably, the inhibitory lineage (nNOS^+^) appeared to be the most severely impacted (Figure 6C), which corresponds to neurons in Branch A.

### The overarching roles of VCL in the neuronal lineage differentiation of ENCCs.

**Figure 7:**
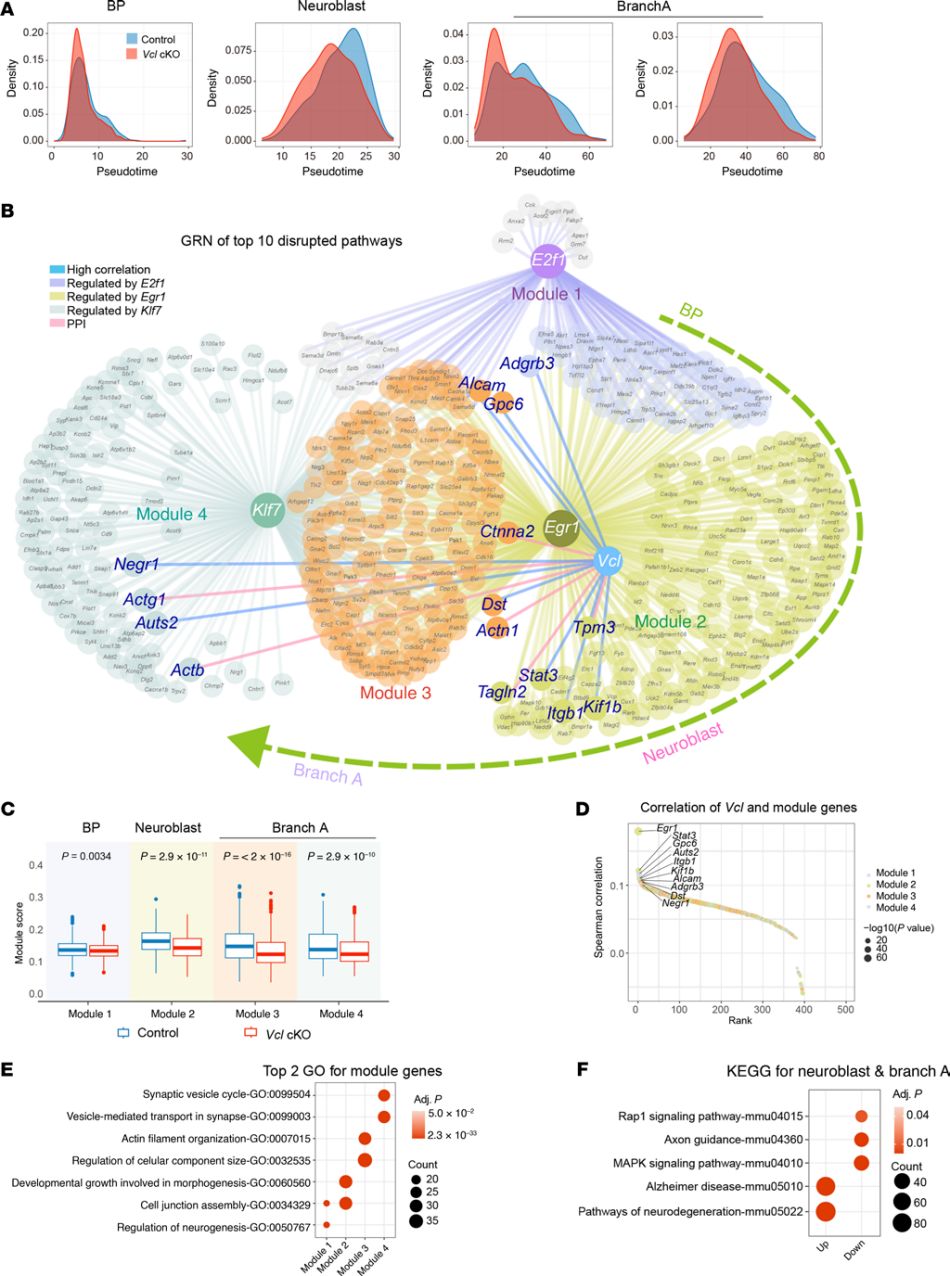
Disrupted gene regulatory network and pathways in BP-to-Branch A lineage. (**A**) The density plot shows the pseudotime distributions of 4 disrupted cell states. E13.5 BP, E15.5 Neuroblast, E13.5 Branch A neurons, and E15.5 Branch A neurons. (**B**) Gene regulatory network of the DEGs along the BP-to-Branch A lineage, colored by regulatory modules defined by regulatory relationship (Module1, genes regulated by E2f1 and Egr1; Module2, genes regulated by Egr1 only; Module3, genes regulated by Egr1 and Klf7; Module4, genes regulated by Klf7 only). Lines connecting *Vcl* represent the potential regulation and known protein-protein interactions from the STRING database. (**C**) The boxplot shows the module score in the corresponding disrupted cell states. (**D**) The dot plot shows the Spearman correlation on the expression of *Vcl* and genes in modules. The top 10 genes ranked by correlation are labeled and shown in **B**. The dot plots show (**E**) the biological processes enriched by the genes in the modules and (**F**) KEGG pathways enriched by the DEGs identified in Neuroblast and BranchA neurons.

We aimed to elucidate the mechanisms responsible for the delayed neuronal differentiation and maturation. To that end, we conducted further analyses on the 4 most impacted cell states: BP at E13.5, Neuroblast at E15.5, and Branch A neurons at both E13.5 and E15.5 (Figure 7A). We identified a total of 3,016 differentially expressed genes (DEGs) (*FDR* < 0.01), which included 35 and 14 consistently downregulated and upregulated genes, respectively, along the BP-to-Branch A trajectory, while 2,967 genes showed a dynamic expression profile. To identify the most biologically relevant DEGs, we employed a 2-tier approach that considered both changes in expression levels and the biological roles of the genes. First, we conducted Gene Ontology (GO) enrichment analysis on the DEGs across each cell state, categorizing them according to biological processes. We then focused on the top 50 GO pathways in each cell state and calculated the corresponding pathway scores for both control and mutant samples (Supplemental Data 4). The 10 most significantly disrupted biological processes within each cell state, comprising 640 DEGs, were selected for further examination based on changes in pathway scores. Subsequently, we examined how these 640 DEGs could influence cellular progression by reclustering them alongside their potential driving genes, specifically transcription factors (TFs), utilizing a gene regulatory network (GRN) inference strategy (Figure 7B and Supplemental Data 5). Among the 640 DEGs, 447 were identified as potential target genes of *E2f1*, *Egr1*, and *Klf7* based on expression correlation and motif binding analyses. These target genes were utilized to construct a GRN, which was organized into 4 distinct modules based on their regulatory relationships. We also recorrelated the genes within these modules with their respective cell states by analyzing the dynamic expression profiles of 3 hub transcription factors: *E2f1*, *Egr1*, and *Klf7*, in control and *Vcl* mutant cells along the differentiation trajectory (Supplemental Figure 8). Notably, *E2f1* exhibited significant differential expression primarily in the BP, while *Klf7* showed disruption specifically in the Branch A neurons. In contrast, expression of *Egr1* decreased consistently across the entire trajectory from BP to Branch A in mutant cells, with the greatest difference from the control cells in Neuroblast (Supplemental Figure 8, A and B). Similar dynamic regulons were also observed consistently (Supplemental Figure 8C). Consequently, we designated Module 1 to BP, Module 2 to Neuroblast, and Modules 3 and 4 to the Branch A neurons (Figure 7C). To assess the impact of *Vcl* loss on these module genes, we then calculated the module scores for both control and mutant cells. The results indicated that all modules exhibited significantly diminished signals in their corresponding cell states in *Vcl* mutants (Figure 7C), suggesting that the absence of *Vcl* disrupts the cellular processes governed by these module genes.

In line with these observations, genes within modules showed stronger expression correlation with *Vcl* compared with other genes (Supplemental Figure 8D). Moreover, VCL demonstrated protein-protein interactions and coexpression relationships with numerous genes across all 4 modules (Figure 7, B and D). These results suggested that VCL mediates the genes in these modules directly or indirectly to govern various cellular processes along BP-to-Branch A differentiation. For instance, integrin β1 (*Itgb1*) within Module 2 is essential for ENCC migration and subsequent ganglionogenesis (20–22). Another gene, transgelin 2 (*Tagln2*), encoding an actin-binding protein that is linked to the migration and proliferation of tumor cells (23), also interacts directly with VCL at the protein level, which may suggest that the cell migration process is affected in *Vcl* mutants. In Module 3, VCL interacts with catenin α-2 (CTTNA)*,* which links cadherin adhesion receptors and the cytoskeleton to regulate cell-cell adhesion and differentiation in the nervous system (24). In addition, Alpha actinin-1 (ACTN1) is a cross-linking protein that interacts with F-actin, playing a crucial role in anchoring actin to various intracellular structures. Morphological changes are essential for initiating neuronal differentiation; thus, VCL likely interacts with these proteins, governing the morphological changes necessary to support subsequent neuronal differentiation.

Additionally, the GO enrichment analysis of the genes within the modules highlighted several disrupted biological processes. Notable findings include neurogenesis within BP (Module 1), cell junction assembly in both BP and Neuroblast modules (Modules 1 and 2), morphology-related regulation and actin filament organization affecting both neuroblasts and Branch A neurons (Modules 2 and 3), and synaptic vesicle function specifically within Branch A neurons (Module 4) (Figure 7E). According to the Kyoto Encyclopedia of Genes and Genomes (KEGG) database, the upregulated genes were associated with neuronal disorders while the significantly downregulated genes were involved in MAPK and Rap1 signaling pathways, which are critical for neuronal differentiation (25) (Figure 7F and Supplemental Data 6). In summary, the results indicate that *VCL* deficiency disrupts multiple cellular processes involved in neuronal lineage differentiation. This disruption begins at the early stages, affecting cell morphogenesis and cell-cell adhesion, which, in turn, impacts the BP and Neuroblast stages. Additionally, the maturation of Branch A neurons is notably hindered, with the MAPK and RAP1 signaling pathways being the most significantly affected. Ultimately, these changes contribute to a delay in the formation of mature Branch A neurons.

**Figure 8:**
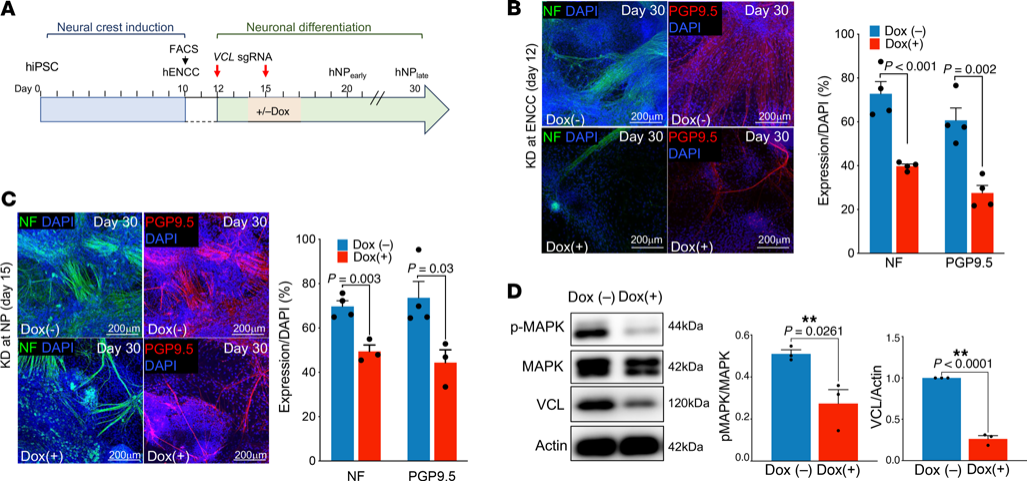
VCL is implicated in multiple stages of neuronal lineage differentiation of ENS cells. (**A**) Schematic shows the human iPSC-based model of ENS development. An inducible-iPSC line was used, where the addition of doxycycline induced the expression of Cas9. Cotransfection of *VCL*-specific gRNA drove the deletion of *VCL* gene in FACS-enriched ENCCs (Day 12) or ENCCs committed to the neuronal lineage (Day 15). Immunocytochemistry with antibodies against pan-neuronal markers (NF and PGP9.5) on cells with *VCL* knockdown on (**B**) day 12 and (**C**) day 15. Bar charts show the percentage of neurons marked by NF or PGP9.5 over the total number of DAPI^+^ cells. Data shown in the bar graphs are mean ± SEM from 3 independent experiments. Student *t* test, 2-tailed. (**D**) Western blot analyses of ITGB1, MAPK, phospho-MAPK and VCL. ACTIN was used as the loading control. The relative expression levels were shown in the bar graphs (mean ± SEM from 3 independent experiments). Student *t* test, 2-tailed. Scale bars: 200 μm.

To evaluate the direct involvement of VCL in the neuronal lineage differentiation of ENCCs, we established an in vitro model using hiPSCs. First, an inducible CRISPR/Cas9–hiPSC (iCas9-hiPSC) line was used to specifically knock down *VCL* in ENCCs or committed neuronal progenitors (NPs), where the expression of Cas9 protein was induced by the addition of doxycycline (DOX) (Figure 8A). A 2-step differentiation protocol was used to model ENCC-to-neuron differentiation. iCas9-hiPSCs were first directed to the NCC lineage by dual-SMAD inhibitors (LDN193189 and SB431542) with a WNT agonist (CHIR99021) and then caudalized with retinoic acid (RA) to obtain posterior/vagal NCCs (hereafter referred to as hENCCs), which were further enriched using FACS and characterized based on the expression of the NC-specific surface markers (HNK1, p75^NTR^, CD49 and SOX10) (Supplemental Figure 9), as described previously (17, 26). The neuronal lineage differentiation of hENCCs was then induced by culturing the hENCCs in neuronal differentiation medium supplemented with various neurotrophic factors (see Supplemental Methods). The *VCL* gene was knocked down in hENCCs or committed NPs by transfecting cells with *VCL*-targeting gRNAs and in the presence of doxycycline on day 12 or day 15, respectively (Figure 8A). By day 30 of differentiation, the neuronal differentiation capacity of the cells was monitored based on the expression of neuronal markers (neurofilament, NF and Protein gene product 9.5, PGP9.5). When *VCL* was knocked down (KD) at NCC stage on day 12, the mutant cells showed a weaker ability to aggregate together, and this severely abolished the subsequent neuronal lineage differentiation. As a result, significantly fewer cells were obtained at day 30 of differentiation in *VCL* KD group, and, among them, there was a lower percentage of cells expressing neuronal markers (NF^+^ or PGP9.5^+^, a marker for clinical diagnosis) compared with the control group (Figure 8B and Supplemental Figure 10A). Similarly, even if we bypassed the neuronal initiation step and KD *VCL* on day 3 after culturing ENCCs in neuronal differentiation medium, the percentage of neurons was significantly lower in the KD group, while the total number of cells was more comparable (Figure 8C and Supplemental Figure 10B). Our scRNA-seq analysis revealed the MAPK pathway as the most significantly disrupted signaling pathway in VCL-deficient cells, particularly in Neuroblast and Branch A neurons. This disruption of the pathway was consistently observed in hENCC-derived neurons when *VCL* was knocked down (Figure 8D). These findings suggest that VCL is essential for the activation of the MAPK pathway, which is crucial for initiating neuronal lineage differentiation of ENCCs and their subsequent maturation.

### Spatial transcriptomic analysis reveals disruption of cell-cell interactions among ENCCs and with the gut mesenchyme in Vcl cKO.

VCL is essential for cell-cell interactions and our scRNA-seq data showed perturbation of integrins and cell junction assembly in *Vcl* mutant ENCCs. Thus, we also examined whether loss of *Vcl* in ENCCs perturbs the communications between ENS cells and their neighborhoods, which may interrupt the ENS development. To this end, we conducted spatial-RNA-seq (ST-seq) analysis on sagittal sections of E13.5 embryos, utilizing replicate tissue sections spaced approximately 16 microns apart. The sequencing of these samples was performed to a median depth of 171,916,081 reads (with an interquartile range of 153,220,340 to 200,680,799), resulting in an average of 6,845 genes and 26,110 unique molecular identifiers (UMIs) per spot (Supplemental Figure 11A). The gut regions were manually delineated based on histological images in each section. We then integrated and jointly analyzed the replicate sections from the control and mutant groups to cover more independent regions of the gastrointestinal tract. Subsequently, deconvolution was employed to estimate the cell type composition for each ST data spot, using a published scRNA-seq dataset of embryonic guts at the same developmental stage as a reference. Through this analysis, ST spots were categorized into specific gut regions according to the relative expression of genes characteristic of the large intestine (*Fxyd4, Muc2, Ntm, Fabp1, Cdx2, Satb2*), small intestine (*Tff3, Tdo2, Lum, Gpr50, Agr2, Sulf1*), and stomach (*Barx1, Sox2, Gata4, Igf1, Nkx2-5*) (Supplemental Figure 11, B and C). Using a similar approach, the spots were further annotated as epithelial (EPI), mesenchymal (MES), and neural crest (NC) cells. Only cells that expressed *YFP* (*YFP^+^*) within these regions were classified as NC (Supplemental Figure 11, D and E).

**Figure 9:**
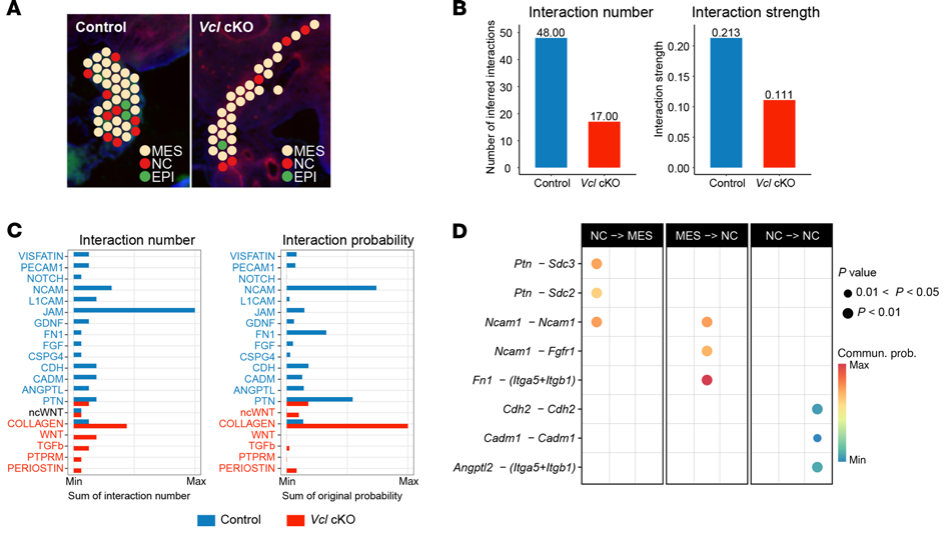
Disrupted cell-cell interactions in *Vcl* cKO revealed by spatial transcriptomics of embryonic guts at E13.5. (**A**) The spatial annotation of the selected regions of the large intestine at E13.5. (EPI, epithelial; MES, mesenchymal cells; NC, neural crest cells.) (**B**) Bar graphs illustrate the total number and strength of NC-related cell-cell interactions. (**C**) The number and strength of NC-related interactions across different pathways are presented. (**D**) The top 3 disrupted cell-cell interactions in each group, ranked by probability. All of these interactions are entirely abolished in the mutant within the corresponding cell-type pairs.

**Figure 10:**
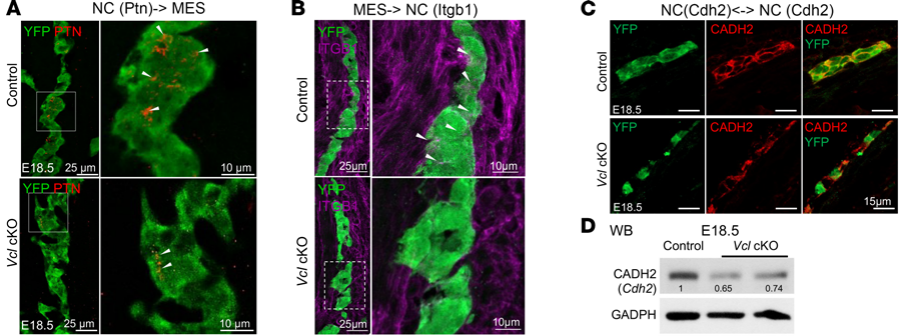
Downregulation of PTN, ITGB1 and CADH2 in *Vcl-*deficient ENS at E18.5. IHC using antibodies against (**A**) PTN; (**B**) ITGB1; and (**C**) N-CADHERIN in the E18.5 distal colon of control and *Vcl* cKO mutants. ENS cells were YFP-labeled. (**D**) A Western blot was conducted with whole gut lysate from control and mutants. The relative CADH2 expression levels are shown compared to the control. GAPDH served as the loading control. Scale bars: 25 μm (**A** and **B**, left); 10 μm (**A** and **B**, right); 15 μm (**C**).

Among the 4 sections, we focused on Control 1 and Mutant 2, which had more comparable numbers of various cell types and covered a larger region of the large intestine compared with the other 2 sections. In particular, the ENS phenotypes of *Vcl* cKO were only observed in the distal colon, so the subsequent analyses were restricted to those regions identified as the large intestine in these 2 sections at similar spatial locations (Figure 9A). Within the selected regions, comparable interactions among EPI and MES were found in the control and mutant (Supplemental Figure 12, A and B), while no common communication between NC and MES was detected. It is likely attributed to the specific deletion of *Vcl* in NCCs, abolishing the communications between NC and their neighboring cells in the mutant. We therefore focused on the interactions within NC and other cell types (Supplemental Data 7). The overall interactions related to NCCs in the *Vcl* mutant exhibited a reduction in both quantity and interaction strength (Figure 9B). Among the 20 interrupted pathways detected, NCAM and PTN pathways exhibited the highest probability of contributing to the reduced interactions found in the mutant (Figure 9C and Supplemental Figure 12C). To further analyze the disrupted cell-cell interactions, we categorized communications by cell-type pairs and identified the top 3 interactions ranked by probability in each cell-type pair (Figure 9D). Among the signal flows from NC to MES, 2 interactions related to PTN (*Ptn-Sdc2/3*) were significantly affected, which aligns with the observed reduction in PTN expression in ENCCs at E18.5 (Figure 10A). The interaction between *Fn1* and the *Itga5+Itgb1* pair was found to be the most impacted signal flow from MES to NC, exhibiting the highest probability. Additionally, a decrease in ITGB1 expression was detected in ENS cells at E18.5 (Figure 10B). Limited NC-NC interactions were detected from the ST data, likely due to the sparse number of NC spots available. Therefore, we investigated NC-NC interactions using scRNA-seq data from E13.5 (Supplemental Figure 13). Notably, we consistently observed reduced Cdh2-Cdh2 interactions in *Vcl* cKO mutants across both the scRNA-seq and ST-seq datasets, alongside diminished N-cadherin (CADH2 encoded by *Cdh2*) expression levels in NC cells (Figure 10, C and D). In summary, *Vcl* deficiency disrupts a crucial signaling pathway PTN (27, 28) and affects adhesion molecules (NCAM and CADH2) (20–22) that are vital for cell-cell interactions, cell migration, and subsequent gangliogenesis. The compromise of these signaling and adhesion molecules impairs cell-cell communication and interactions between NC and neighboring cells, ultimately hindering the development of the ENS.

### Loss of Ptn perturbed the formation of circumferential smooth muscle cell layer in Vcl cKO and Ptn KO.

**Figure 11:**
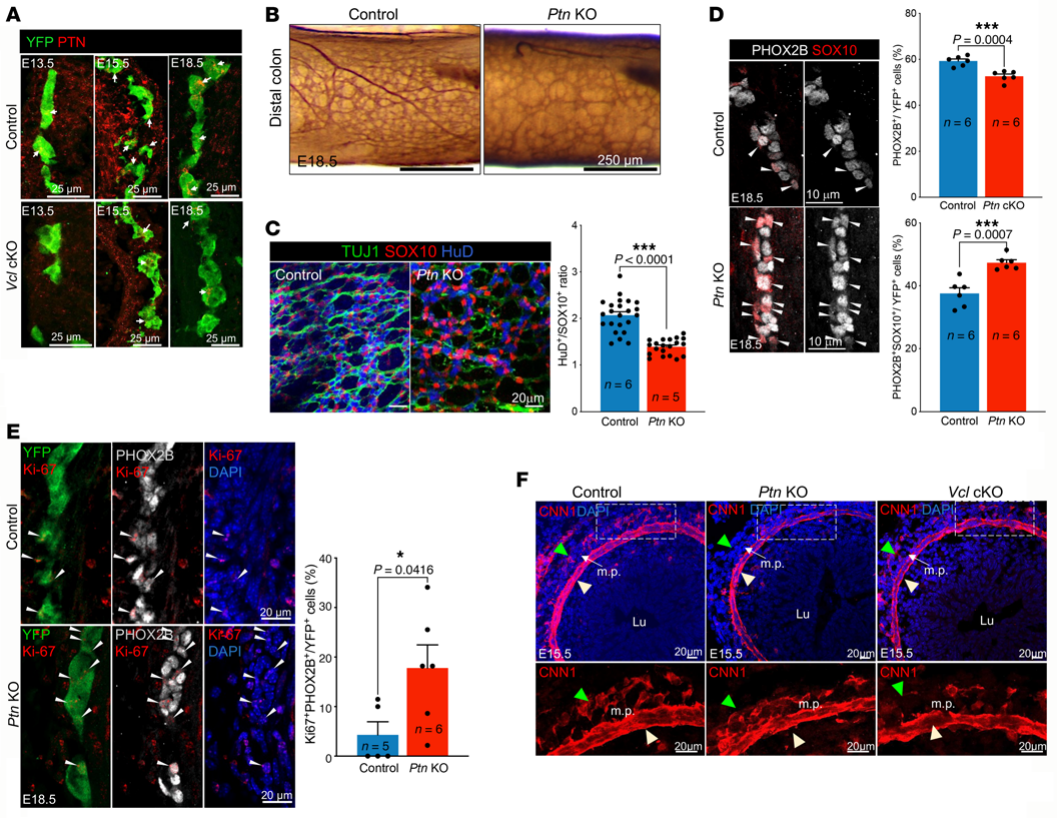
Dynamic expression of PTN in ENS and mesenchyme is essential for the formation of the muscle layer of the gut. (**A**) IHC of PTN in E13.5, E15.5 & E18.5 of control (*Wnt1-Cre; Rosa26^YFP^*) and *Vcl* cKO (*Vcl* KO*^Wnt1–Cre^*) guts. (**B**) Whole-mount acetylcholinesterase (AchE) staining of control and *Ptn* KO colon. (**C**) Whole-mount immunofluorescence of TUJ1, HuD, and SOX10 shows ENS network aberrantly organized in the distal colon of E18.5 *Ptn* mutant. The average neuron-to-glia ratio in the 4–5 selected regions of distal colon is shown in the bar graphs. Immunofluorescence of (**D**) PHOX2B and SOX10 and (**E**) Ki67 counterstained with DAPI on cross sections of E18.5 distal colon. (**F**) Immunofluorescence staining for Calponin (red) and counterstained nuclei with DAPI (blue) in E15.5 Control (*Ptn^+/+^*) and *Ptn^−/−^* colons. m.p., myenteric plexus; Lu, lumen; lamina muscularis interna (yellow arrowhead) and the lamina muscularis externa (green arrowhead). Bar graphs show the quantitative data (mean ± SEM; n, number of embryonic guts analyzed, *P < 0.05; ***P < 0.001, Student *t* test, 2-tailed). Scale bars: 25 um (**A**); 250 um (**B**); 20 um (**C**, **E**, and **F**); 10 um (**D**).

Our spatial RNA-seq data revealed multiple disruptions in cell-cell communication between ENCCs and MES cells in the *Vcl* cKO colon. While the functions of NCAM and CADH2 in the ENS are well established, the role of PTN remains less defined. Our findings suggest that PTN is the most significantly affected pathway mediating communication between ENCCs and MES cells. Further expression analysis demonstrated that PTN is expressed in both ENCCs and gut MES cells; notably, its expression in ENCCs increases with developmental stages, whereas in MES cells, the highest expression is found at E15.5 (Figure 11A and Supplemental Figure 14). Notably, both ENCCs and MES cells exhibited downregulation of PTN in *Vcl* cKO mutants across all developmental stages examined. We next sought to understand the biological functions of PTN signal in ENS and gut development. The *Ptn^–/–^* mutants consistently exhibited ENS defects resembling the *Vcl* cKO mutants, but with less severity. They included hypoganglionic colon (Figure 11B), a disorganized ENS network of reduced neuron-to-glia ratio (Figure 11, C and D) and immature ENS with reduced percentage of postmitotic neurons (Figure 11E). Intriguingly, PTN is essential for the smooth muscle lineage differentiation of MES cells, which contribute to the formation of the 2 circumferential smooth muscle layers of the colon: the lamina muscularis interna and lamina muscularis externa. *Ptn^–/–^* mutants at E15.5 exhibited a thinner lamina muscularis interna and a delayed spatial separation between the 2 smooth muscle layers. A similar phenotype was observed in *Vcl* cKO mutants, where only a few Calponin-positive (CNN1-positive) smooth muscle cells were present in the lamina muscularis externa at this stage (Figure 11F). This phenotype likely results from impaired smooth muscle differentiation.

## Discussion

VCL is a critical molecular component involved in mediating cellular interactions with the extracellular environment. To date, only 3 *VCL* mutations have been reported. L954del and R975W were found to be associated with either sporadic or familial dilated cardiomyopathy. These 2 mutations specifically affect the tail region of the VCL protein, potentially disrupting the binding of VCL with actin and paxillin (29, 30). The M209L mutation, on the other hand, located in the head region of VCL protein, was later identified in a syndromic patient who presented with HSCR disease and congenital cardiac defects (17). Through reanalysis of our in-house sequencing dataset combined with functional validation, we identified 4 loss-of-function mutations in the *VCL* gene in 7 patients with HSCR. Notably, the M209L mutation was identified in 3 patients with syndromic short-segment HSCR exhibiting both ENS and cardiac anomalies. This finding prompted further investigation into the role of VCL in neural crest development and its implications in neurocristopathies.

In mice, we found that NC-specific deletion of *Vcl* has a severe impact on the development of the NCCs. The mice exhibited cardiac outflow tract and ENS defects mirroring the patients with syndromic HSCR. *Vcl* cKO ENS exhibited hypoganglionosis or partial aganglionosis in the distal colon. Structurally, the hypoganglionic region resembles the transition zone of the HSCR bowel, characterized by a mixture of abnormal ganglion cells and nerve structures. This area is also distinguished by a thicker muscularis externa layer and a comparatively thinner muscularis interna relative to the ganglionic region (31). In E18.5 embryos, mutant colons consistently exhibited a reduced quantity of meconium, indicative of potential dysmotility within the mutant gastrointestinal tract. *Vcl* cKO died a few hours after birth, precluding a more detailed analysis in the postnatal ENS.

The colonic hypoganglionosis of *Vcl* cKO was likely attributed to defects not only in cell-cell interactions but also in neuronal differentiation of ENCCs. Loss of cell-cell interaction caused only a slight delay in gut colonization, but in conjunction with immature neuronal differentiation, it significantly disrupted ganglion formation, thereby greatly perturbing the establishment of the ENS network in the bowel. As observed in short-segment HSCR patients, the distal colon of the mutant was the most affected, with many more immature or partially committed ENS neurons residing in the distal colon. It raises the possibility that these partially committed ENS neurons remain migratory and migrate along with the wavefront cells, reaching the distal colon. The lack of coordination between migration and differentiation, along with disrupted cell-cell interaction, interfered with ganglion formation, leading to colonic hypoganglionosis.

VCL and integrins (10), as well as the roles of integrins in mediating gut colonization by ENCCs (32), are well documented. However, the influence of VCL on the neuronal lineage differentiation of ENCCs remains unclear. To address this, we conducted single-cell transcriptomic analyses of ENS cells isolated from E13.5 and E15.5 embryonic guts, stages during which distinct differentiation trajectories are established. Unlike abolishing a specific signaling pathway associated with cardiac neural crest defects (33), VCL appears to have a more prominent role in regulating morphological changes that promote neuronal differentiation and support neuron maturation through interactions with the cytoskeleton. The dysregulation of the MAPK pathway was identified as a major factor contributing to differentiation defects beyond morphological abnormalities. This pathway acts as a central signaling cascade integrating multiple key signals involved in ENS development, including the GDNF and ERBB pathways. Although some disruptions were observed in certain ERBB pathway genes, these were limited to 2 subpopulations. Notably, most *VCL* carriers lack the *RET* variant, suggesting that *VCL* may be a RET-independent gene associated with HSCR; however, additional studies with larger patient groups are needed to confirm this hypothesis. VCL is implicated in various stages of neuronal lineage progression, spanning from BP to neuroblast to Branch A neurons. It influences critical processes, such as cell adhesion and cytoskeletal dynamics, which are essential for morphological modifications and axonogenesis, ultimately contributing to a delay in cell advancement along the developmental trajectory if lost. Consistent with in vivo observations, using the hiPSC-based model, inducible deletion of *VCL* before and after the start of differentiation also prevented the neuronal lineage from forming, highlighting the role of VCL in early stages of ENS development.

In addition to the cell-autonomous effect, the loss of VCL in ENCCs severely interfered with the interactions among ENS cells and their neighbouring (MES) cells, as revealed by spatial transcriptomic analysis. Down-regulation of N-cadherin (CADH2) disrupts cell-cell interactions among ENCCs, which may explain the presence of solitary ENS cells at the migratory front of the mutant gut and retarded ganglion formation. Intriguingly, we identified PTN as a key regulator in the signaling pathway between ENCCs and gut mesenchymal cells. In the central nervous system, PTN modulates processes such as cell migration, neural progenitor proliferation and self-renewal, neurite outgrowth, and synaptic plasticity through its interaction with multiple receptors, including syndecan (SDC) (34), and the subsequent activation of critical signaling cascades such as c-SRC, PI3K, and ERK1/2 (35). Although PTN has previously been regarded as a growth factor secreted by the gut mesenchyme (36), its specific functions within the ENS have remained poorly understood. In this study, we revealed a role for PTN, demonstrating that it not only influences ENCC differentiation but also functions as an ENCC-secreted factor that mediates gut mesenchyme development, contributing to the formation of the 2 distinct muscle layers of the bowel. Based on our data, we found no significant correlation in expression between these *Vcl* and *Ptn* genes (Figure 7D). The reason for the downregulation of PTN in the MES of *Vcl* cKO remains unclear. Considering that elevated PTN expression occurs in mature enteric neurons and that its expression is influenced by various growth factors (37, 38), we speculate that the immature status of ENS neurons and MES may contribute to the reduced PTN expression observed. Alternatively, its changes could be the result of the loss of cell-cell interactions in *Vcl* cKO. Abnormal muscle layers in HSCR bowel suggest ENS might support muscle development (31), but little is known about the specific molecules and signals guiding these cellular interactions during gut development. Therefore, further research is necessary to elucidate the specific roles of PTN in the ENS and muscle development, as well as to understand its regulatory mechanisms.

In summary, we identified VCL as a hub gene coordinating the interactions between ENS and MES that are essential for gut development. VCL modulates multiple signaling pathways involved in ENS development, and it exerts a subtle and continuous influence across various stages of ENS formation. Despite notable differences between humans and mice, which may impact mutation penetrance, the mouse still represents the best in vivo model for studying disease mechanisms. Here, we showed that the accumulation of minor disruptions caused by the loss of VCL ultimately leads to a phenotype characterized by partial aganglionic or hypoganglionic bowel, similar to features seen in short HSCR. These subtle changes in signaling pathways and cell processes may represent a common, overarching mechanism underlying the pathogenesis of short HSCR.

## Methods

### Sex as a biological variable.

Our study examined male and female patients and mice, and similar findings were observed in both sexes.

### Patients.

We reanalyzed our in-house whole-genome and whole-exome sequencing datasets (18, 19, 39) to identify potential mutation(s) affecting *VCL*. A total of 94 patients with sporadic (no family history) HSCR with short-segment aganglionosis were included in this study. An in-house program, KGGSeq (40), was used to assess the variant pathogenicity, which made use of valuable biological resources and knowledge, providing a comprehensive and efficient framework to filter and prioritize genetic variants from whole-exome and whole-genome sequencing data.

### Construct construction.

The full-length, head, and tail domains of human *VCL* (NM 00373) were obtained by PCR with specific primers (Supplemental Table 1) and subcloned into pCMV-GFP (Addgene, Plasmid #11153), pFLAG-CMV2 expression vector (Sigma), and pCMV-Myc-N (Clonetech), respectively. HSCR-associated mutations were then introduced into VCL expression constructs using the QuikChange Lightning Site-Directed Mutagenesis Kit (Agilent Technologies) with specific primers listed in Supplemental Table 1 according to the manufacturer’s protocol. The WT and mutated *VCL* expression constructs were subsequently transfected into HeLa cells and subjected to immunofluorescent and pull-down assays.

### Mice.

*Wnt1-Cre* (strain# 022137), *Rosa26^YFP^* (strain# 006148) and *Vcl^f/f^* (strain# 028451) mice were purchased from Jackson Lab. *Ptn* heterozygous knocKOut (T028403) mice were purchased from GemPharmatech.

### Generation of an iCas9-hiPSCs.

To generate a hiPCS line with an inducible expression of Cas9, the inducible cassettes, one containing the iCas9 coding sequence (41) under the regulation of the tight TRE promoter and the other carrying the M2rtTA tetracycline response element (42), were sequentially targeted into the AAVS1 loci by CRISPR-Cas9D10a nickase-mediated homologous recombination with 2 gRNAs (Supplemental Table 1). It was followed by selection with puromycin and neomycin, and the properly targeted neomycin and puromycin-resistant clones were selected using PCR. The inducible expression of Cas9 was validated by Western blot.

### Derivation of ENCC and NPs from iCas9- iPSC lines.

A dual SMAD-inhibition protocol was used to generate hENCCs from hiPSC. The hENCCs were enriched by FACS with antibodies against p75^NTR^ and HNK-1 at day 10 of the differentiation. The FACS-enriched hENCCs were then cultured in neuronal differentiation medium for 10 and 20 days to generate early and late NPs, as described (17, 26, 43). *VCL* was deleted either on ENCC or at day 4 of differentiation by the addition of 2 μg/ml doxycycline and followed by transfection of *VCL*-targeting gRNAs.

### Co-IP & Western blotting.

co-IP was performed as previously described (44). In brief, cell lysates were incubated with anti-GFP or anti-FLAG M2 Magnetic Beads (Sigma, M8823) overnight at 4°C. Antibody/protein complexes were washed with lysis buffer for four times and analyzed by Western Blotting. For Western blotting, protein lysates were separated on 8%–12% SDS-polyacrylamide gels and blotted onto nitrocellulose membranes. The membranes were then incubated with primary and secondary antibodies (Supplemental Tables 3 and 4).

### AChE stain.

The Acetylcholinesterase Rapid Staining Kit (MBL) was used to stain the ENS following the manufacturer’s protocol.

### Immunofluorescence studies.

Cells, whole mounts, and sections of embryonic guts were fixed in 4% PFA, incubated in blocking buffer followed by primary and secondary antibodies (Supplemental Table 3 and 4), and counterstained with DAPI. For whole-mount staining, a clearing step was included by incubating the samples in Murray’s Clear solution as described previously (44). All immunofluorescence images were captured using Carl Zeiss LSM 800 or LSM900 confocal microscope.

Carl Zeiss LSM 800 was also used for live imaging where Z-stack images were captured every 5 minutes for 12–15 hours as described previously (45). Immunofluorescence images and live imaging were processed and analysed using ImageJ (NIH) and Chemotaxis and Migration Tool plugin (ibidi), respectively.

### Droplet-based scRNA-seq and spatial RNA-seq.

The FACS-sorted cells were then subjected to droplet-based scRNA-seq using Chromium Single Cell platform and Single Cell 3’ Library Kits (10x Genomics). Spatial transcriptomics was carried out using the 10x Genomics Visium platform with Visium Spatial Gene Expression Slides & Reagent kits with four sagittal sections from control and mutant. Details of the bioinformatic analysis are included in the Supplemental Methods.

### Statistics.

Individual data points per mouse (5–6 animals per genotype) and mean ± SEM are displayed in the figures. Data were analyzed using an unpaired 2-tailed Student’s *t* test or 1-way ANOVA. *P* < 0.05 was considered significantly different. Statistical analyses and data visualization were performed using Prism 10. In scRNA-seq data analysis, Empirical Bayes moderated *t* test implemented in Scanpro was used for the cell type proportion comparison, with Benjamini-Hochberg-adjusted *P* < 0.05 considered significant. Two-sided unpaired Wilcoxon rank-sum test was used to compare the expression level and module score, where *P* < 0.05 considered significantly different. Two-sided asymptotic 2-sample Kolmogorov-Smirnov test was used to compare the pseudotime distributions. Bonferroni-adjusted *P* < 0.01 was considered significantly different.

The experimental details can be found in Supplemental Methods.

### Study approval.

For the human study, informed consent was obtained from all participants and the study was approved by the institutional review board of the University of Hong Kong and the Hospital Authority ((HKU/HA HKW IRB) UW 13-225). All animals were kept in the Animal Laboratory of the University of Hong Kong, and all experiments were performed in accordance with procedures approved by the Committee on the Use of Live Animals, the University of Hong Kong (CULATR 23-493 and CULATR 23-029).

### Data availability.

Raw sequencing data for E15.5 and E13.5 mouse ENS from the scRNA-seq and the ST-seq data are deposited in the Sequence Read Archive under BioProjects PRJNA1295088, PRJNA752243 and PRJNA1006077, respectively. Processed data and code are accessible on Zenodo (https://doi.org/10.5281/zenodo.15979367) and GitHub (https://github.com/ellylab/Vcl-ENS). Public datasets used in this article can be downloaded from Gene Expression Omnibus using accession numbers GSE149524 and GSE186525. All data points shown in graphs are available in the Supporting Data Values file.

## Author contributions

LL and ML performed bioinformatics analyses under the supervision of ESWN and AJ, respectively. XW analyzed the mouse mutants. PHYC and KKYW provided the clinical data. MMGB conducted the genetic analyses. PL and CYY performed in vitro studies. ESWN supervised the whole project. ESWN and LL prepared the manuscript.

## Funding support

- Seed funding from the University of Hong Kong.
- GRF grants from the Research Grants Council of Hong Kong Special Administrative Region, China Hong Kong (HKU17111824 &17107322) to ESWN.

## Supplementary Material

Supplemental data

Supplemental data set 1

Supplemental data set 2

Supplemental data set 3

Supplemental data set 4

Supplemental data set 5

Supplemental data set 6

Supplemental data set 7

Unedited blot and gel images

Supplemental video 1

Supporting data values

## Figures and Tables

**Table 1:**
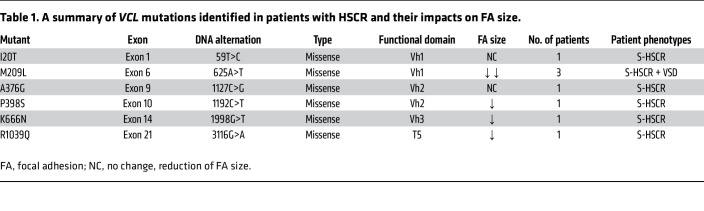
A summary of *VCL* mutations identified in patients with HSCR and their impacts on FA size.

## Version 1 12/09/2025

In-Press Preview

## Version 2 02/02/2026

Electronic publication
