# Supplementary material for: Vinculin influences essential processes in enteric nervous system development and Hirschsprung disease pathogenesis
Source: J Clin Invest. 2025 Dec 9;136(3):e198531. doi: 10.1172/JCI198531 (PMC12867144; doi:10.1172/JCI198531)
Supplement: Supplemental data [file jci-136-198531-s108.pdf]

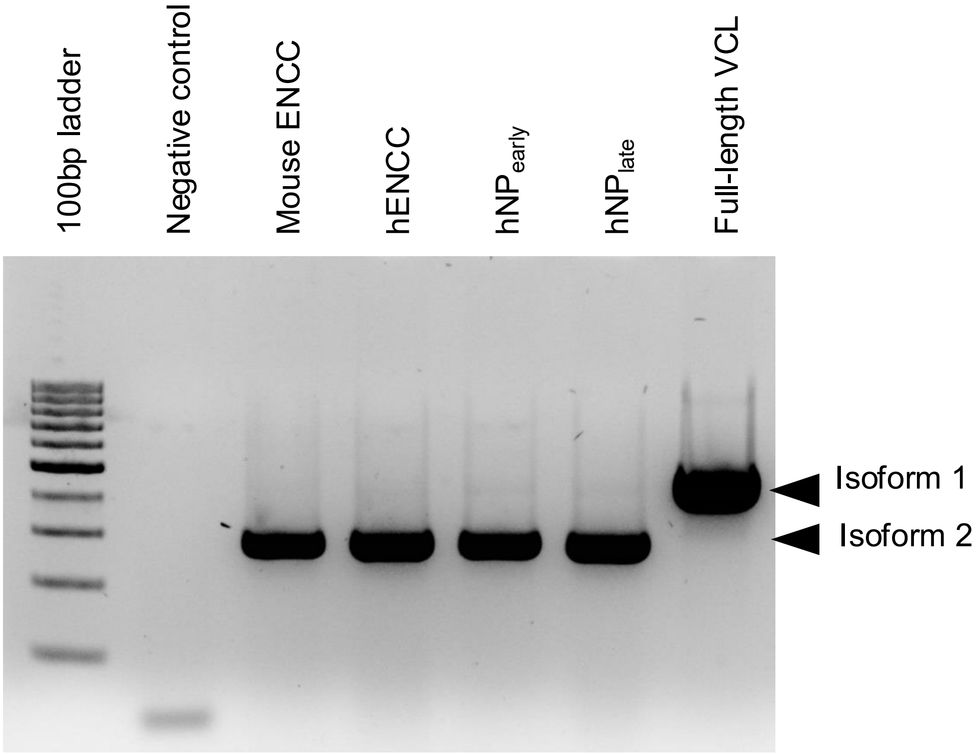

Supplementary Figure 1. Expression of *VCL* isoform 2 in mouse and human ENCCs. RT-PCR data show the expression of *Vcl* isoform 2, but not isoform 1, in mouse ENCCs, human iPSC-derived ENCCs, and their neuronal derivatives (hNP$_{early}$ and hNP$_{late}$: days 9 and day 20 of neuronal differentiation).

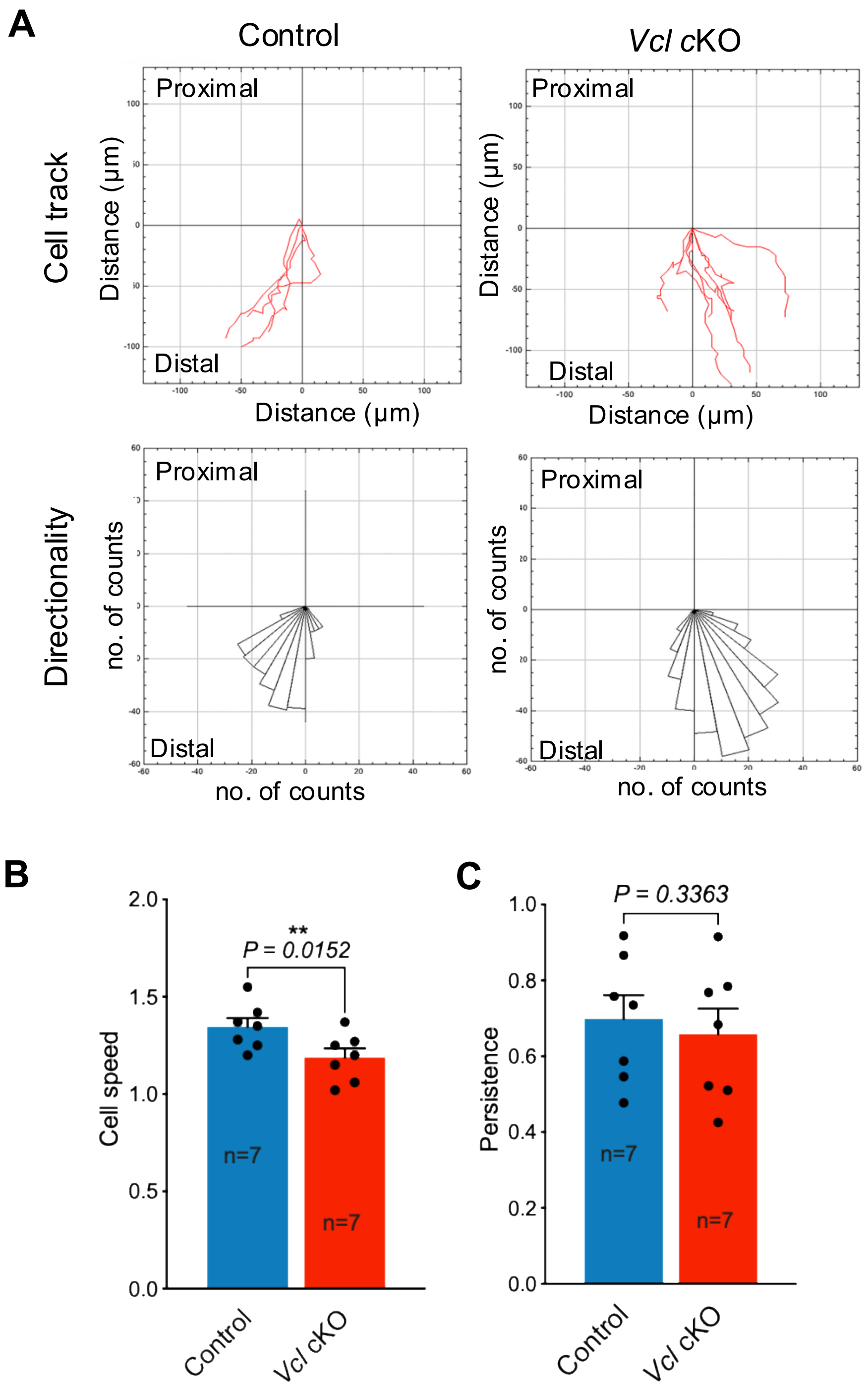

Supplementary Figure 2. Regular migratory trajectory and pattern found in *Vcl* cKO (**A**)Trajectory plot (upper panel) and polar histograms (lower panel) represent the trajectories of the most caudal cell at 10-minute intervals in 3 explants of E12.5 hindgut. ENCCs in both *Vcl* cKO and control showed a uniform tendency to migrate distally. Bar plots show the **(B) c**ell speed at which the migratory wave front of ENCCs migrated caudally along the gut and **(C)** persistence of control and *Vcl* null ENS cells.

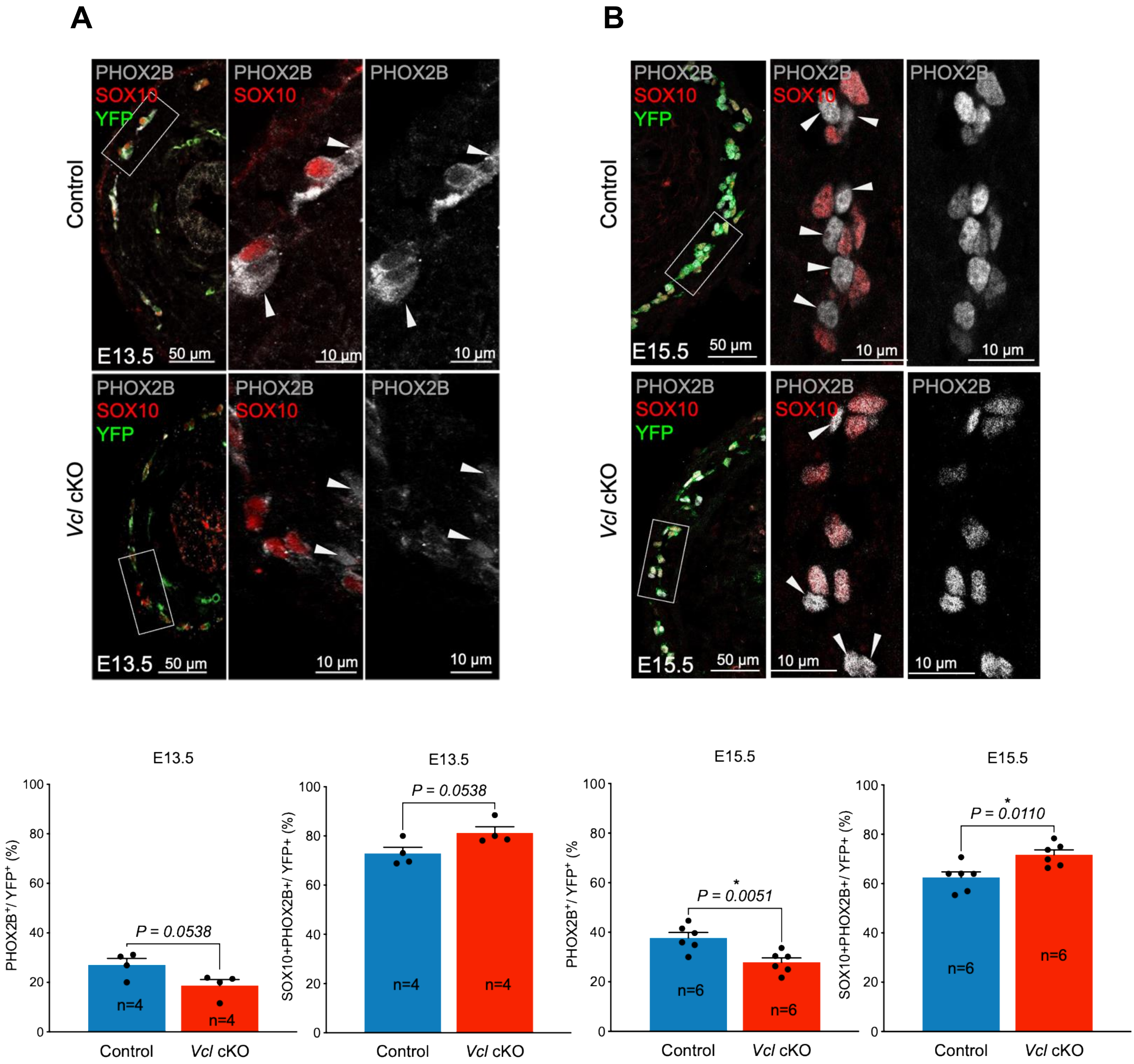

Supplementary Figure 3. Delayed neuronal differentiation of ENCCs in *Vcl* cKO at E13.5 & E15.5. Immunoflurosecence of Phox2b and Sox10 in control (*Wnt1-cre; Rosa26*^{YFP}) and mutant (*Wnt1-cre;Rosa26*^{YFP}*;Vclf/f*) at **(A)** E13.5 and **(B)** E15.5. All ENCCs were labeled with YFP. Bar charts display the quantitative data on the percentages of ENCCs committed to the neuronal lineage (Phox2b+, marked by arrow heads) and the uncommitted (Phox2b$^+$Sox10$^+$) ENCCs. (mean ± SEM, n: number of embryonic guts analyzed, *P*<0.05 was considered significantly different, student *t*-test, two-sided).

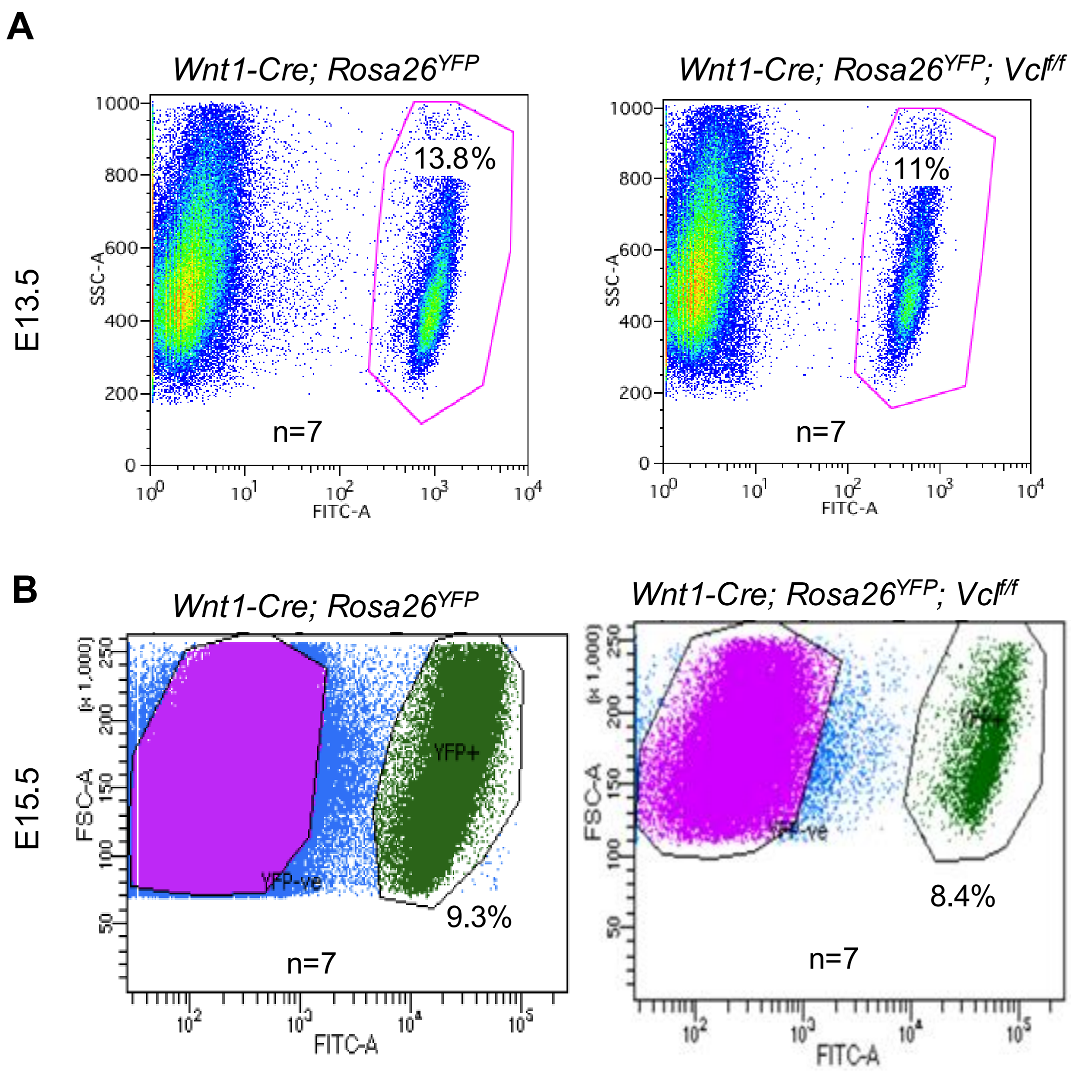

Supplementary Figure 4. Enrichment of ENCCs from the control and *Vcl* cKO guts at E13.5 & E15.5 by FACS. Seven enzyme-dissociated embryonic guts from 2-3 litters were subjected to FACS. The scatter plots show comparable percentages of YFP+ ENCCs were isolated from control and mutant embryonic guts at **(A)** E13.5 and **(B)** E15.5.

QC of scRNA-seq data

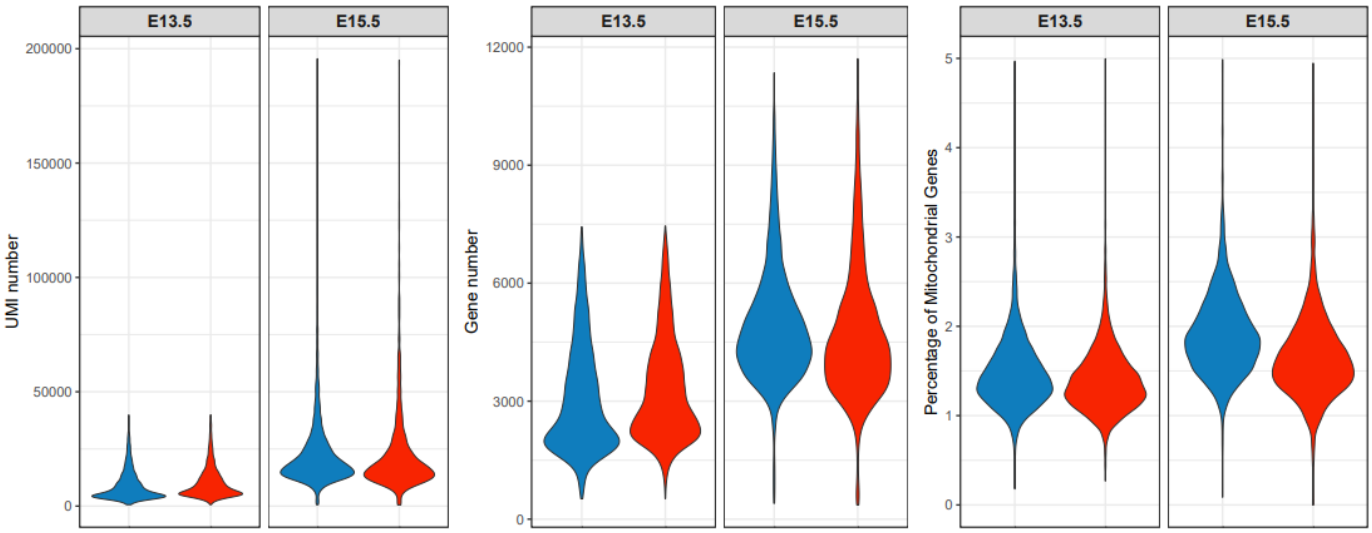

Supplementary Figure 5. Quality control of scRNA-seq data. Violin plots displaying (left) the number of unique molecular identifiers (UMIs) per cell, (middle) the number of detected genes per cell, and (right) the percentage of mitochondrial gene per cell.

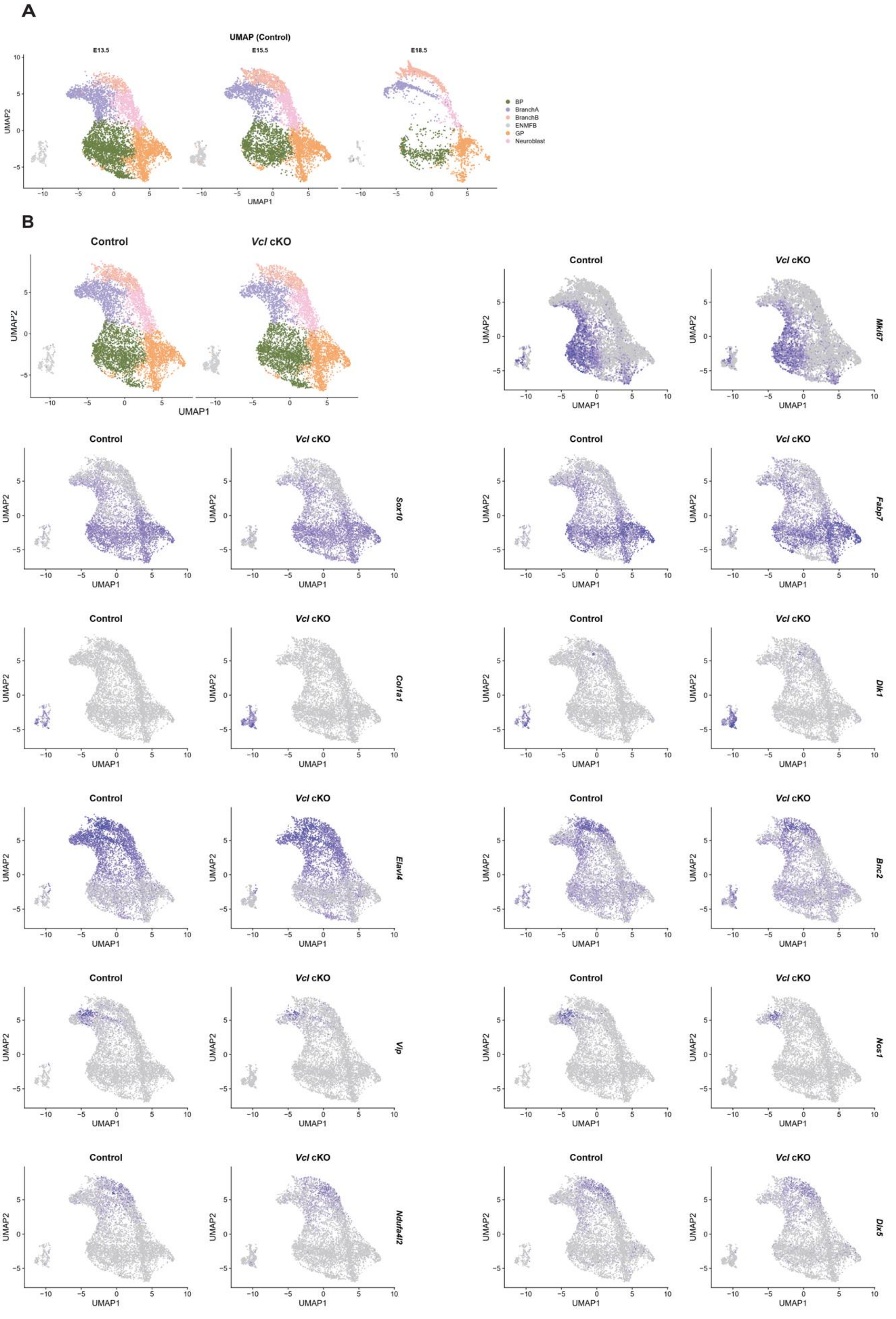

Supplementary Figure 6. Annotation of scRNA-seq data. (A) Integrated UMAP projection of control scRNA-seq data at E13.5, E15.5, and E18.5 from Mikhailova et al. 2021**. (B)** Expression of selected marker genes and proliferative markers in the control and mutants.

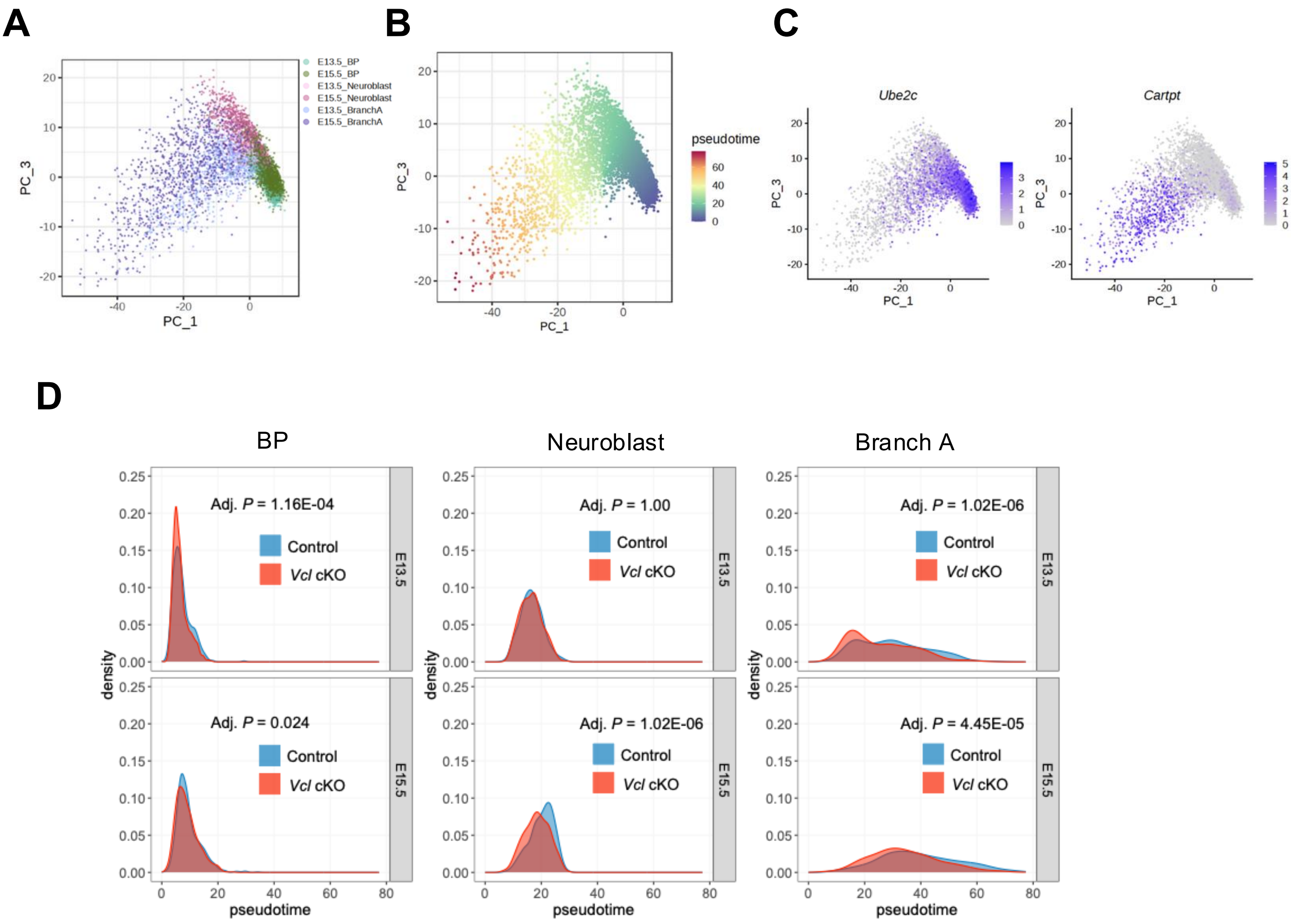

Supplementary Figure 7. Trajectory construction using cells in disrupted cell states. **(A)** Cell distribution in a low-dimensional embedding of PCA1 and PCA3. **(B)** Inferred pseudotime of cells across disrupted cell states. **(C)** Scaled expression of selected marker genes for BP (*Ube2c*) and Branch A neurons (*Cartpt*) in the PCA plot. **(D)** Density plot shows the pseudotime distributions across disrupted cell states. Two-sided asymptotic two-sample Kolmogorov-Smirnov test.

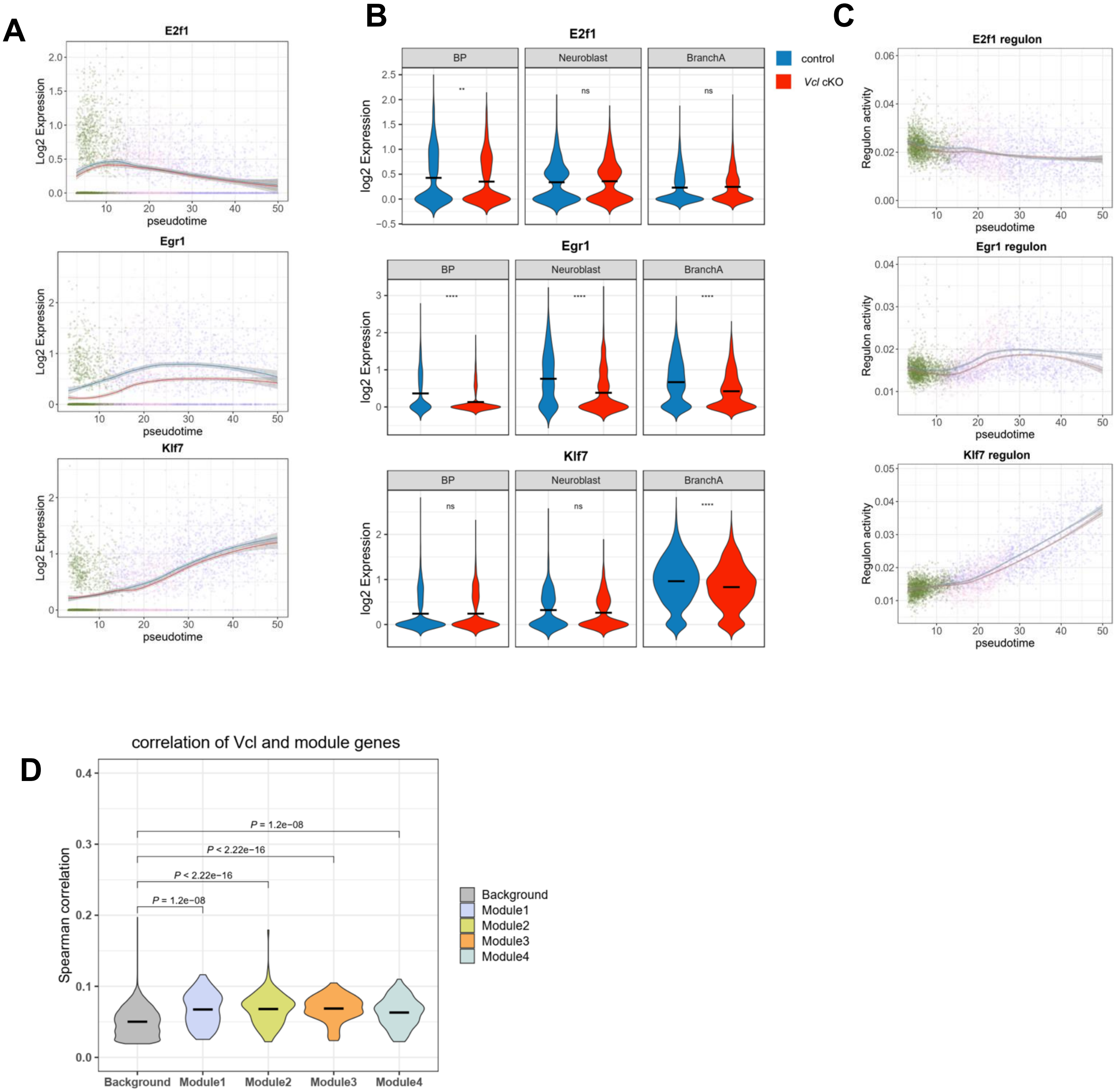

Supplementary Figure 8. Dynamic changes of TFs along trajectory. (A) Expression of TFs along the trajectory. **(B)** Expression of TFs in different cell states. **(C)** Regulon activity along the trajectory. **(D)** Spearman correlation of *Vcl* and genes in modules.

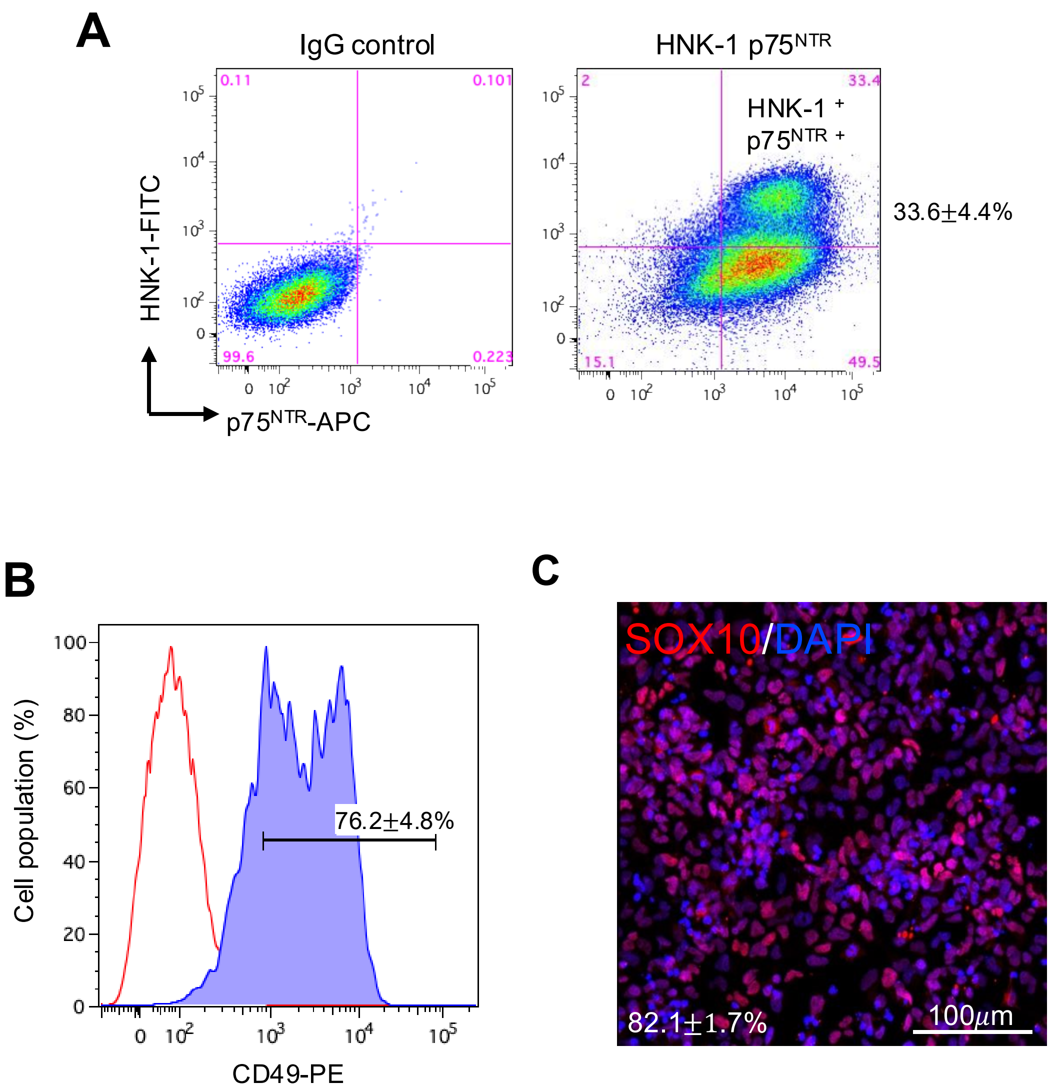

Figure S9. Enrichment and characterization of hiPSC-derived ENCCs. ENCCs were identified and enriched on Days 10 of neural crest induction based on their expression of neural crest markers(HNK-1, p75NTR and CD49) using fluorescent-activated cell sorting (FACS). IgG control was used as a negative control. **(A)** Flow cytometry dot plots show the percentages of cells expressing HNK-1 and $p75^{NTR}$ in each group. HNK-1 and $p75^{NTR}$ double-positive cells were enriched by FACS. **(B)** The majority of HNK-1 and $p75^{NTR}$ double-positive cells (76.2 ± 4.8%) co-express CD49. **(C)** Immunocytochemistry with antibody against SOX10 is performed in HNK-1 $^{+}$ and $p75^{NTR +}$ cells.

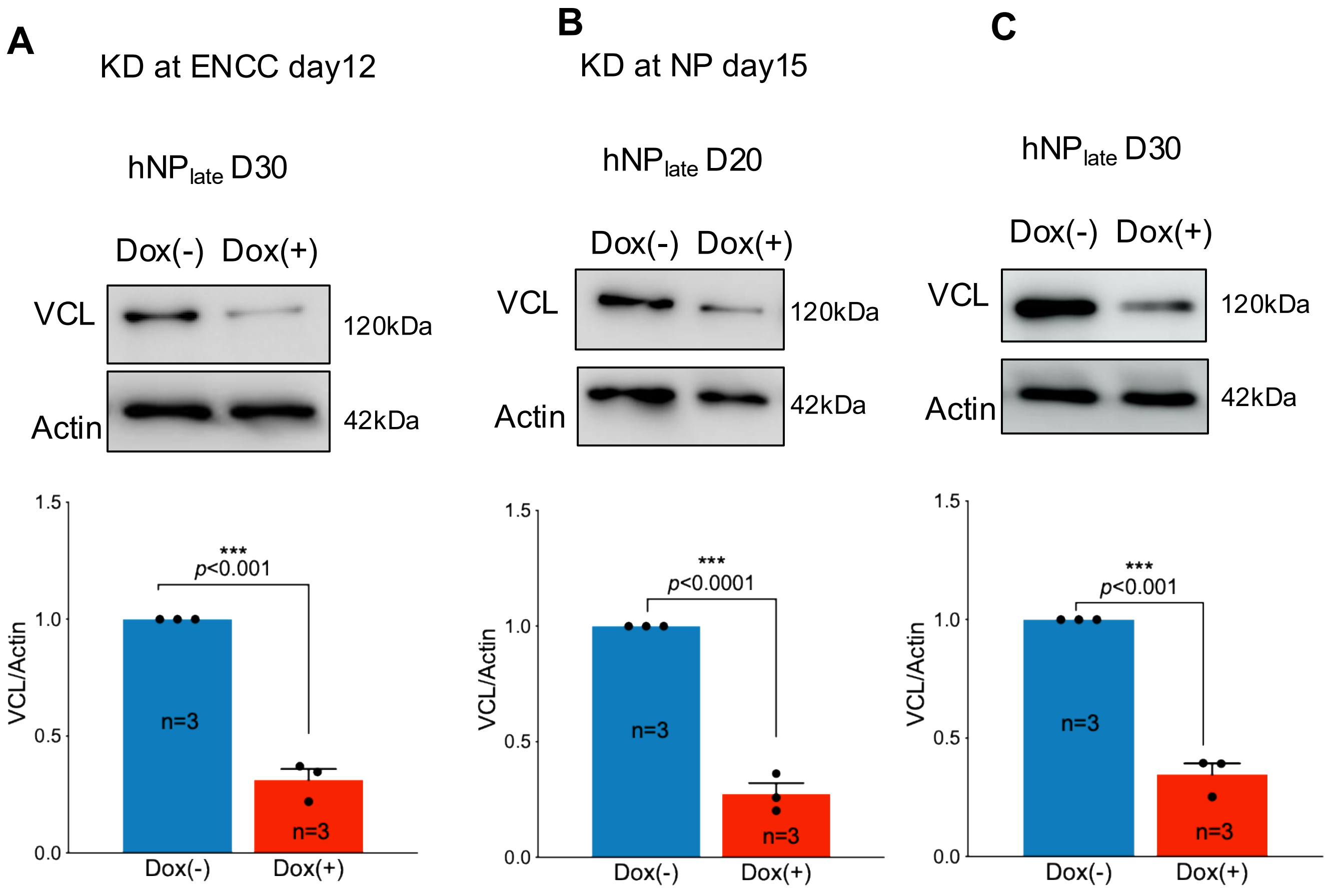

Supplementary Figure 10. Western blots of VCL expression. *VCL* was knocked down in **(A)** ENCCs on Day 12 and **(B & C)** in NP on Day 15. Expression of VCL was detected by Western blotting on Days 20 and 30 of neuronal differentiation, as shown. A representative image from each set is shown. Bar charts illustrate the relative expression of VCL in control and KD cells (mean ± SEM) from three independent experiments. ACTIN was used as the loading control. A *P*-value of <0.05 is considered statistically significant, based on a two-sided Student's *t*-test.

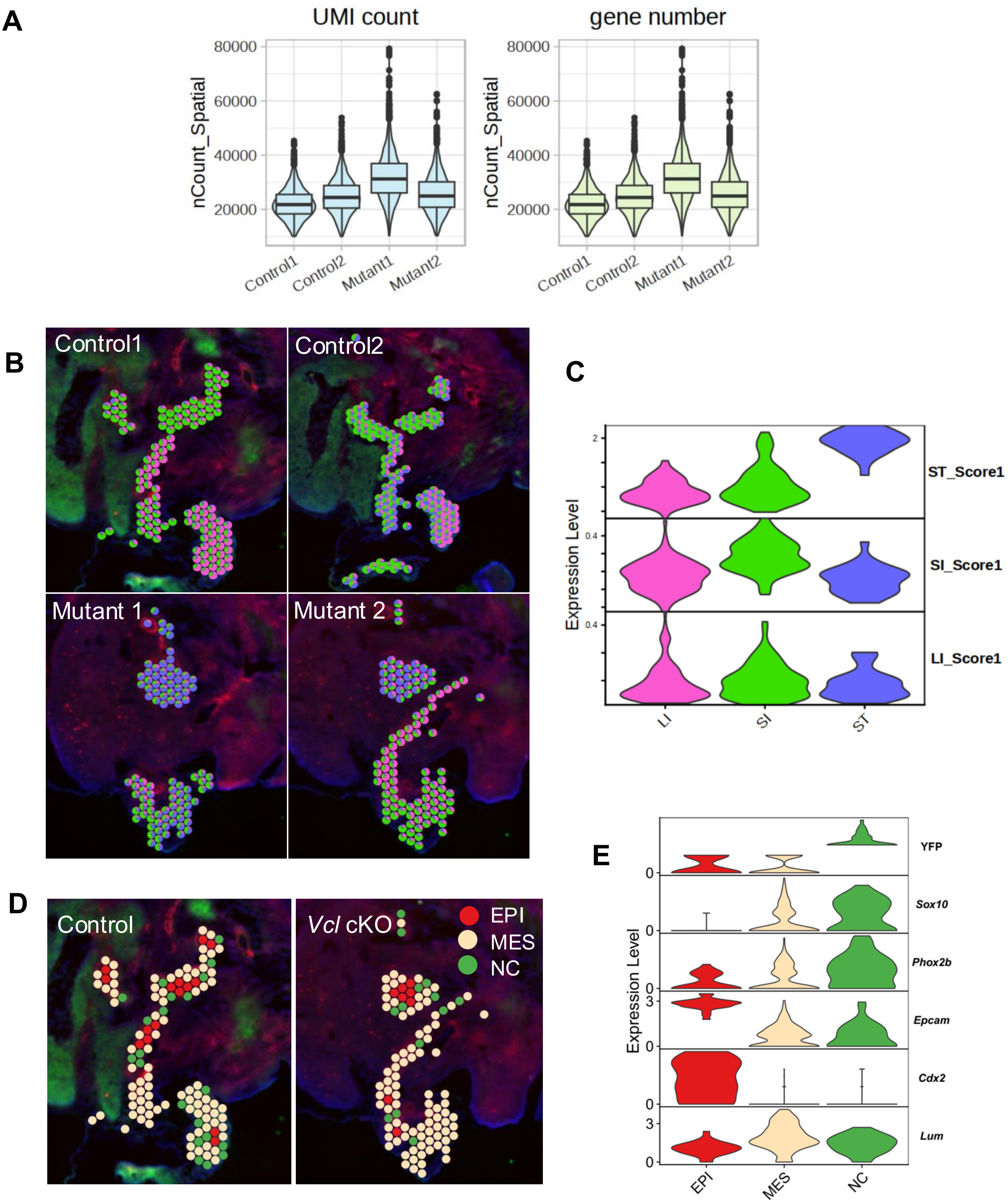

Supplementary Figure 11. Quality control and spatial annotations for gut region at E13.5. (A) The UMI count (nCount_Spatial) and the number of detected gene number (nFeature_Spatial) of 4 datasets of cells from 4 sections of E13.5 control and mutant embryos. **(B)** Spatial scatter pie plot displays inferred region composition in each spot in the gut region. LI, large intestine; SI, small intestine; ST, stomach. **(C)** Violin pot showed the module scores calculated using a set of marker gene of different gut regions. **(D)** Representative images of spatial annotation of all spots using Control 1 and Mutant 2, which comprised comparable numbers of various cell types. Hereafter, they are labeled as Control and *Vcl* cKO, respectively. EPI, epithelium; MES, mesenchymal cells; NC, neural crest cells. **(E)** Violin plot shows the expression of selected marker genes of EPI, MES and NC.

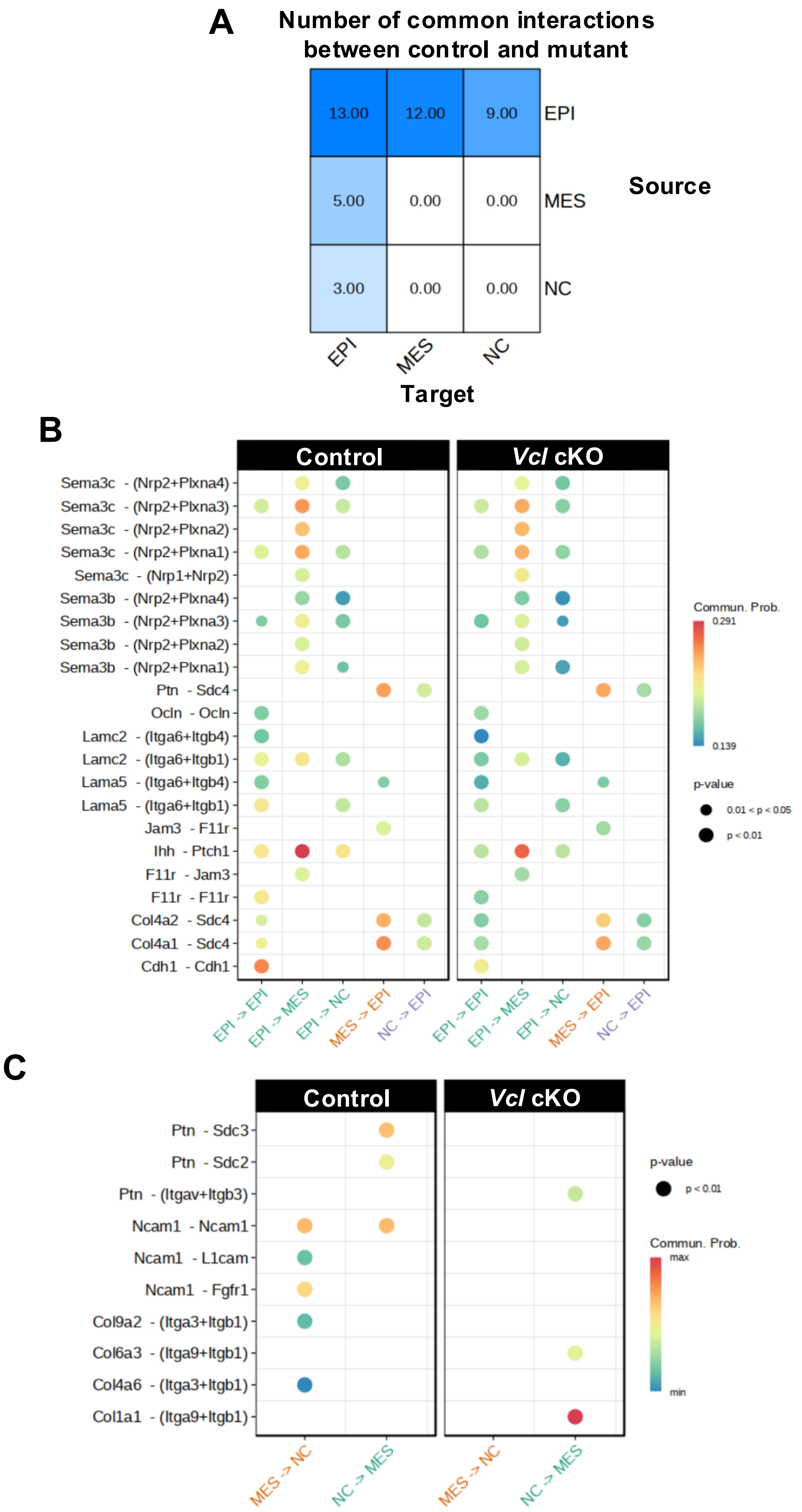

Supplementary Figure 12. Inferred cell-cell interactions in E13.5 Visium data. (A) The heatmap showed the number of common cell-cell interactions occurring between identical cell-type pairs in control and *Vcl* mutant. **(B)** Dot plot shows the common cell-cell interactions in (A). **(C)** The cell-cell interactions in top two signaling pathways of control and mutant, ranked by the total interaction probability as shown in Figure 6C, with only those *P* value < 0.01 displayed.

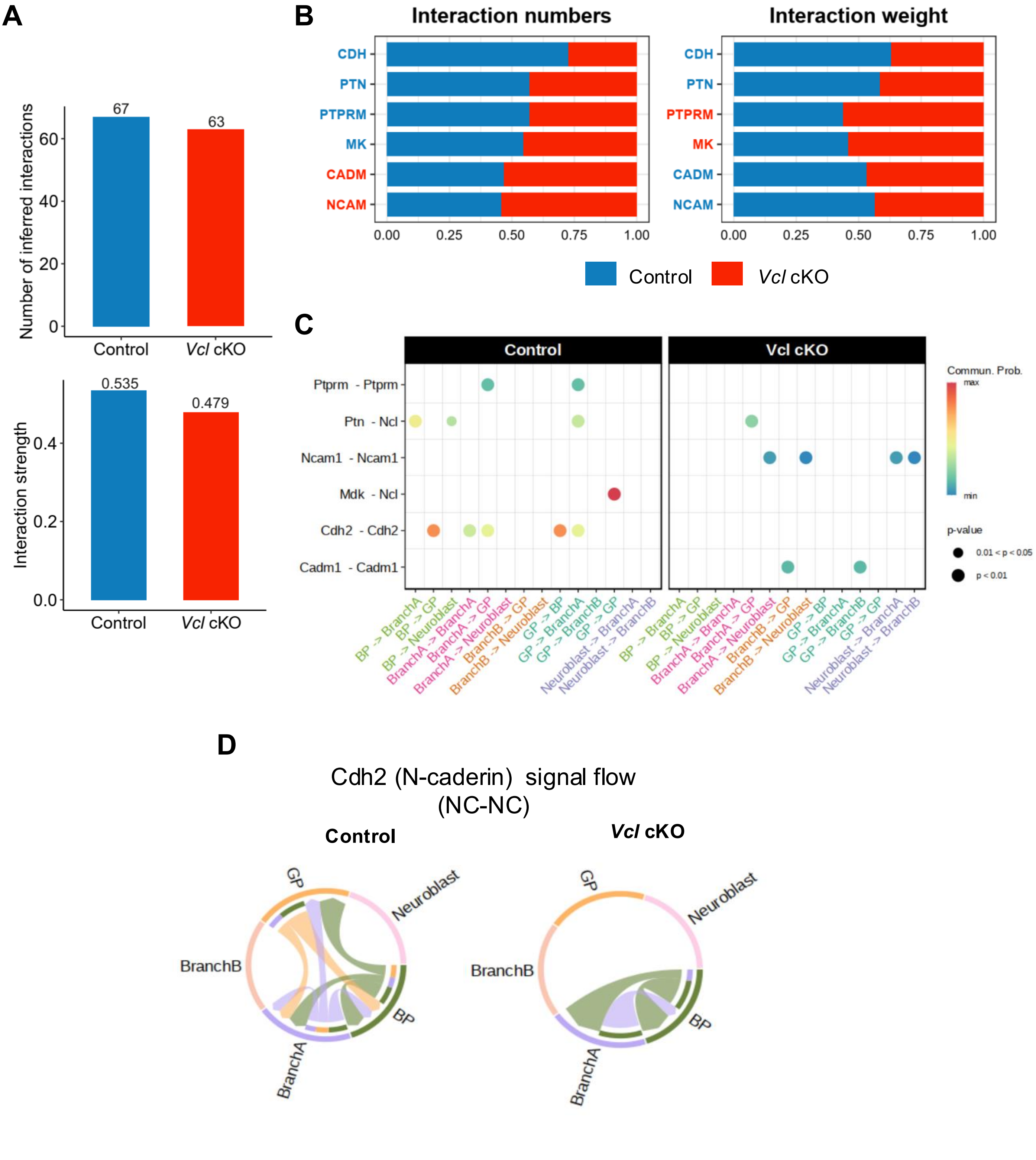

Supplementary Figure 13. Inferred cell-cell interactions using scRNA-seq data at E13.5. (A) Total number and strength of cell-cell interactions inferred using E13.5 scRNA-seq data. **(B)** Total number and probability of cell-cell interactions across different signaling pathways. **(C)** The disrupted cell-cell interactions identified as gained or lost in *Vcl* mutant compared to the control. **(D)** Chord diagram shows the signal flow of N-caderin among different cell states.

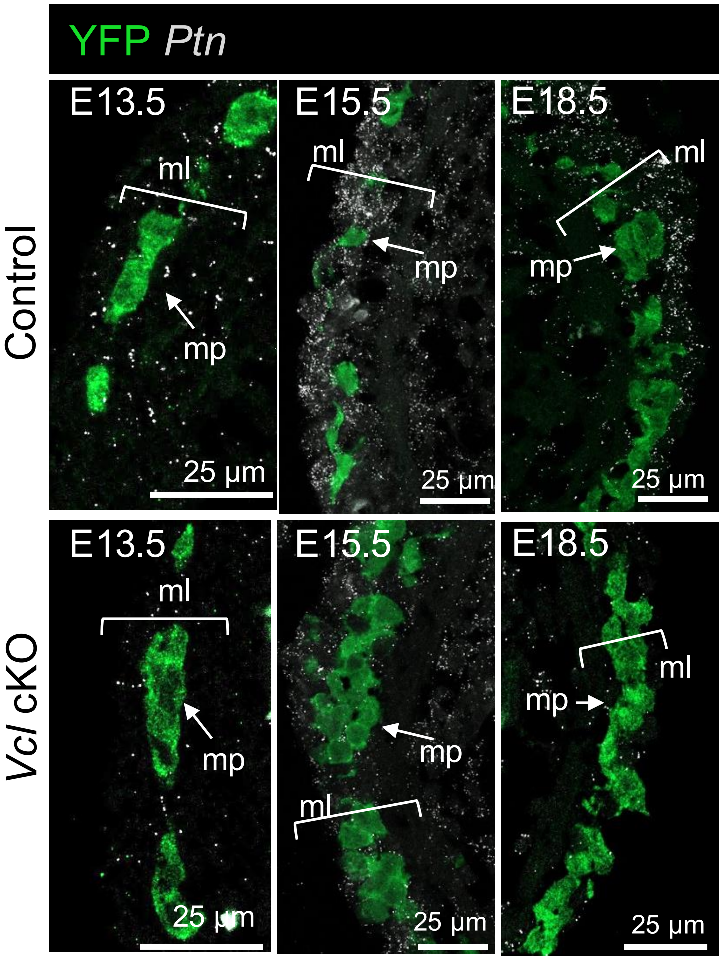

Supplementary Figure 14. Dynamic expression of *Ptn* in ENCCs and gut mesenchyme. RNAscope detected the *Ptn* transcripts in E13.5, E15.5 and E18.5 embryonic guts in control and *Vcl* cKO.

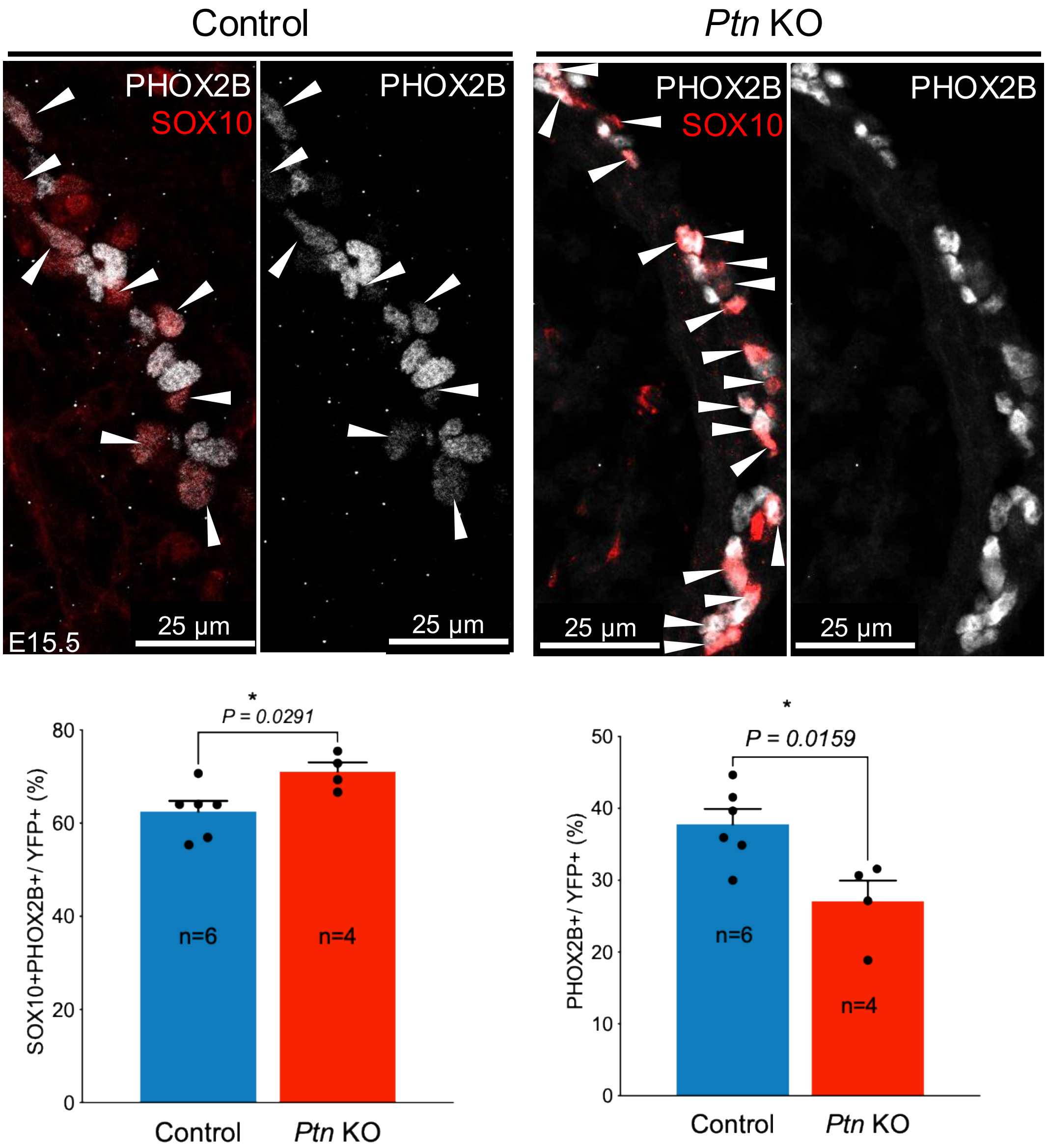

Supplementary Figure 15. Delayed neuronal differentiation of ENCCs in *Ptn* KO at E15.5. Immunoflurosecence of Phox2b and Sox10 in control (*Ptn*$^{+/+}$) and mutant (*Ptn*$^{-/-}$) at E15.5. Bar charts display the quantitative data on the percentages of ENCCs committed to the neuronal lineage (Phox2b+) and the uncommitted (Phox2b^{+}Sox10^{+}, marked by arrow heads) ENCCs. (mean$\pm$SEM, n: number of embryonic guts analyzed, P<0.05 was considered significantly different and marked by "*", student *t*-test, two-sided).

Supplementary Table 1: List of oligos for gene targeting.

Genes/Allele	Orientation	Sequence (5' to 3')	Applications
hVCL full length	Forward	ATGCCAGTGTTTCATACGCGCACG	Generation of VCl expression vector
	Reverse	CTACTGGTACCAGGGAGTCTT	
hVCL-I20T	Forward	GGCACAGCAGaccTCCCACCTGG	Generation of Vcl-I20T
	Reverse	ACCGGCTCCAGGATGCTC	
hVCL-M209L	Forward	GTTGCTGCCAGTTCTCATTTCAGCTttgAAG ATTTTTGTAACAAC	Generation of Vcl-M209L
	Reverse	CCAGCTTCATCTAAGATCTG	
hVCL-A376G	Forward	CAAGCTGGAAggcATGACCAACT	Generation of VCL-A376G
	Reverse	CGAGCTGCATTTTCCACTTTTG	
hVCL-P398S	Forward	GCTTGCAGATtcaAATGGTGGAC	Generation of VCL-P398S
	Reverse	CAGTTCTGAGCAGCATCG	
hVCL-K666N	Forward	GGCCTCAGTGaatACGGCCCGAG	Generation of VCL-K666N
	Reverse	TGAATGCCTTCCACTGTTGATTTATTAGC	
hVCL-R1039Q	Forward	GGAGACTGTGcagGAAGCTGAAG	Generation of VCL-R1039Q
	Reverse	TTCACAGACTGCATGAGG	
hVCL-A977P	Forward	GCATCGGGAAcctACCAAGTGGTCTAGTAAGG	Generation of VCL-A977P
	Reverse	AAGGACTGAGCCGCGGCC	
hVCL Head Domain (aa1-258)	Forward	CTGGAATTCAATGCCAGTGTTTCATACGCG	Generation of Flag-Vcl head plasmid
	Reverse	CTGTCTAGACCAGGCATCTTCATCCCA AG	
hVCL Tail Domain (aa879-1066)	Forward	CTGGAATTCGGGAGGAAAAGGATGAAGAGTTC	Generation of Myc-Vcl tail plasmid
	Reverse	CTGCTCGAGACTACTGGTACCAGGGAGTC	
AAVS1 (1)	Forward	TTTCTTGGCTTTATATATCTTGTGGAAAGGACGAAACACCGTCACCAATCCTGTCCCTAG	Generation of iCas9-hiPSC line
	Reverse	GACTAGCCTTATTTTAACTTGCTATTTCTAGCTCTAAAACCTAGGGACAGGATTGGTGAC	
AAVS1 (2)	Forward	TTTCTTGGCTTTATATATCTTGTGGAAAGGACGAAACACCGCAGTGGGGCCACTAGGGAC	
	Reverse	GACTAGCCTTATTTTAACTTGCTATTTCTAGCTCTAAAACGTCCCTAGTGGCCCCACTGC	
VCL	Forward	CCACCTGGTGATAATGCACG	Generation of *VCL* KO ENCCs
	Reverse	GCTCGAGATTATCTAATTGA	

Remarks: mutated sequences are in small cap; the targeted sequences are underlined.

Supplementary Table 2: List of PCR primers for genotyping.

Genes/Allele	Orientation	Sequence (5' to 3')	Product size
Cre transgene	Forward	CGTACTGACGGTGGGAGAAT	374 bp
	Reverse	TGCATGATCTCCGGTATTGA	
Vcl$^{flox/flox}$	Forward	CATCATGAGTTCTTGACCTGGA	443 bp/550 bp
	Reverse	TGCAAACCCTAATAATTTTACGAA	
Ptn$^{-/-}$	Forward	CACACACACACACACACTATTGTTCACT	422bp/293bp
	Reverse	TGACACTGTAACAGTGTAACATTCTTGG	
	Forward	TCTAGGCATCTTTCATCTGGTAAAGGG	
	Reverse	CCCGGATGTCGCCTTATACAGTCA	
Rosa26^{YFP}	W-forward	CTGGCTTCTGAGGACCG	384 bp/184 bp
	W-reverse	CAGGACAACGCCCACACA	
	YFP-forward	AGGGCGAGGAGCTGTTCA	
	YFP-reverse	TGAAGTCGATGCCCTTCA	

Supplementary Table 3: List of primary antibodies used in this study

Primary antibody	Target antigen	Dilution	Assays	Category #	RRID
Mouse-anti-Tuj1	Neuronal classIII β-tubulin	1:500	IHC	Biolegend (801202)	AB_10063408
Rabbit-anti-Tuj1	Neuronal classIII β-tubulin	1:200	ICC	Abcam (ab18207)	AB_444319
Mouse-anti-HuD	ELAV Like RNA Binding Protein 4	1:200	IHC	Santa Cruze (SC-28299)	AB_627765
Rabbit-anti-nNOS	nNOS	1:100	IHC	Thermo Fisher Scientific (617000)	AB_2313734
Mouse-anti-Sox10 (CL4455)	SOX (SRY-related HMG-box) family transcription factors Sox10	1:100	ICC	Atlas Antibodies (AMAb91297)	AB_2665884
Goat-anti-Phox2b	Phox2b	1:100	IHC	R&D system AF4940	AB_10889846
Anti-alpha SMA	α-smooth muscle actin	1:500	IHC/ICC	Abcam ab21027	AB_1951138
Mouse-anti-Ki67 Clone B56	Human Ki67	1:100	IHC	BD Pharmingen 556003	AB_396287
Goat anti-Integrin beta 1/CD29	Recombinant mouse Integrin beta 1	10 μg/ml	IHC	R&D Systems AF2405	AB_416591
Rabbit anti-Vinculin	Vinculin	1:100	IHC	Abcam ab91459	AB_2050446
Mouse anti-Vinculin Clone hVIN-1	Vinculin	1:300	WB	Sigma-Aldrich V9131	AB_477629
Mouse anti-N-cadherin Monoclonal Antibody (3B9)	Cdh2, N-cadherin	1:100/1:1000	IHC/WB	Thermo Fisher Scientific 33-3900	AB_2313779
Rabbit anti-PTN antibody-C-terminal	Pleiotrophin	1:100	IHC	Thermo Fisher Scientific Cat# PA5-145351	AB_3094082
Rabbit anti-Cleaved caspase3	Activated caspase3 with cleavage adjacent to Asp175	1:100	IHC	Cell Signaling 9661	AB_2341188
Sheep anti-GFP	Green fluorescent protein and Yellow fluorescent protein	1:200	IHC	Bio-Rad 4745-1051	AB_619712
Chicken anti-GFP	Green fluorescent protein and Yellow fluorescent protein	1:200	IHC	Thermo Fisher Scientific A10262	AB_2534023
Rabbit anti-GFP antibody - ChIP Grade	GFP-tag	1:500	IP	Abcam ab 290	AB_303395
Chicken-anti-Neurofilament-Light (NF)	Neurofilament	1:200	ICC	Neuromics (CH22105)	AB_2737102
Rabbit-anti-PGP9.5	Protein gene product 9.5	1:200	ICC	Abcam (ab108986)	AB_10891773
Mouse anti-CRISPR-Cas9 Clone 7A9-3A3	Cas9	1:1000	WB	Abcam ab191468	AB_2692325

Rabbit anti-p44/42 MAP Kinase	p44/42 MAP Kinase	1:1000	WB	Cell Signaling Technology 9102	AB_330744
Mouse anti-Phospho-p44/42 MAPK	Phospho-p44/42 MAPK (Thr202/Tyr204)	1:1000	WB	Cell Signaling Technology 9106	AB_331768
Mouse anti-Actin, C4	Actin	1:20000	WB	Millipore MAB1501	AB_2223041
Mouse anti-FLAG M2	FLAG	1:10000	WB	Sigma-Aldrich F1804	AB_262044
Anti-FLAG M2 Magnetic Beads	Anti-FLAG M2 Magnetic Beads		IP	Sigma-Aldrich M8823	AB_2637089
Rabbit anti-c-Myc	c-Myc	1:1000	WB	ABclonal A19032	AB_2862524
Rabbit anti Myc-Tag pAb-C-terminal	Myc-Tag pAb-C-terminal	1:500	IP	ABclonal AE009	AB_2771925
Mouse anti-Paxillin, clone 5H11	Paxillin	1:2000	ICC/WB	Millipore 05-417	AB_309724
Rabbit-anti-Phospho-Paxillin (Tyr118) Polyclonal Antibody	Phospho-Paxillin (Tyr118)	1:1000	WB	Thermo Fisher Scientific PA5-143737	AB_2942965
CD271-FITC	Neurotrophin receptor $p75^{NTR}$	1:100	Flow cytometry	Miltenyi Biotec 130091917	AB_871651
HNK1-APC	CD57	1:100	Flow cytometry	BD Biosciences 560845	AB_10563760
ITGA4-PE	PE/Cyanine7 Anti-human CD49d	1:100	Flow cytometry	BioLegend 304314	AB_10643278

Supplementary Table 4. Secondary antibodies used in this study

Secondary antibody	Dilution	Applications	Category #	RRID
Donkey anti-Sheep IgG (H+L) Cross-Adsorbed Secondary Antibody, Alexa Fluor™ 488	1:500	IHC	Invitrogen A-11015	AB_2534082
Alexa Fluor® 488 Donkey Anti-Chicken IgG H&L	1:500	IHC	Jackson ImmunoResearch 703-545-155	AB_2340375
Donkey anti-Mouse IgG (H+L) Highly Cross-Adsorbed Secondary Antibody, Alexa Fluor™ 488	1:500	IHC	Invitrogen A-21202	AB_141607
Donkey anti-Rabbit IgG (H+L) Highly Cross-Adsorbed Secondary Antibody, Alexa Fluor™ 488	1:500	IHC	Invitrogen A-21206	AB_2535792
Donkey anti-Mouse IgG (H+L) Highly Cross-Adsorbed Secondary Antibody, Alexa Fluor™ 594	1:500	IHC	Invitrogen A-21203	AB_2535789
Donkey anti-Rabbit IgG (H+L) Highly Cross-Adsorbed Secondary Antibody, Alexa Fluor™ 594	1:500	IHC	Invitrogen A-21207	AB_141637
Donkey anti-Mouse IgG (H+L) Highly Cross-Adsorbed Secondary Antibody, Alexa Fluor™ 647	1:500	IHC	Invitrogen A-31571	AB_162542
Donkey anti-Rabbit IgG (H+L) Highly Cross-Adsorbed Secondary Antibody, Alexa Fluor™ 647	1:500	IHC	Invitrogen A-31573	AB_2536183
Donkey anti-Goat IgG (H+L) Cross-Adsorbed Secondary Antibody, Alexa Fluor™ 647	1:500	IHC	Invitrogen A-21447	AB_2535864
Goat-anti-rabbit HRP	1:2000	WB	Dako Cytomation (P0448)	AB_2617138
Goat-anti-mouse HRP	1:2000	WB	Dako Cytomation (P0447)	AB_2617137

Materials and Methods

Patients

We reanalyzed our in-house whole-genome and whole-exome sequencing datasets(1-3) to identify potential mutation(s) affecting *VCL*. A total of 94 sporadic (no family history) HSCR patients with short-segment aganglionosis were included in this study. These patients are Chinese and were consecutively recruited at Queen Mary Hospital, where they underwent surgery. Seventy-two patients were male and twenty-two were female. Twelve patients (three females and nine males) had associated anomalies in the heart, including ductus arteriosus (PDA), atrioventricular septal defect, and bicuspid aortic stenosis. Another twelve male and seven female patients had other defined syndromes such as Down syndrome, Ondine's Curse, and Wolff-Parkinson-White (WPS) syndrome. The remaining patients had no associated anomalies (isolated HSCR). HSCR diagnosis was based on histological examination of either biopsy or surgical resection material for the absence of enteric plexuses. Informed consent was obtained from all participants and the study was approved by the institutional review board of the University of Hong Kong and the Hospital Authority ((HKU/HA HKW IRB) UW 13-225).

We used one program which integrates all predictions made by other programs: Variant pathogenecity was assessed by an in-house program KGGSeq(4). KGGSeq is a software platform that consists of bioinformatics and statistical genetics functions, making use of valuable biological resources and knowledge, providing a comprehensive and efficient framework to filter and prioritize genetic variants from whole-exome and whole-genome sequencing data. Importantly, KGGSeq integrates "knowledge" resources from epigenetic databases, biological pathways and protein-protein interaction networks to annotate the genes that harbour any post-QC variants as well as to predict the potential pathogenicity of their variants. For the later, KGGSeq integrates 4 prediction programs (Polyphen2, Sift, MutationTaster and Likelihood ratio) which are weighted by logistic regression.

Those rare variants predicted deleterious, unique to the patients analyzed and whose MAF was zero or <1% in public databases (The 1000 Genome Project, the NIH Exome Sequence Project of 6500 individuals -ESP6500-) or in-house exome sequencing projects (N=900 Chinese individuals) were selected for validation. Thus, Sanger sequencing of the patients' DNA was performed to validate the next-generation sequencing data. Sanger sequencing of parental DNA was also performed (when available) to assess the origin of the variant.

Construct construction

The full-length, head, and tail domains of human *VCL* (NM 00373) were obtained by PCR with specific primers (Supplementary Table 1) and subcloned into pCMV-GFP (Addgene, Plasmid #11153), pFLAG-CMV2 expression vector (Sigma), and pCMV-Myc-N (Clonetech), respectively. HSCR-associated mutations were then introduced into VCL expression constructs using the QuikChange Lightning Site-Directed Mutagenesis Kit (Agilent Technologies, Santa Clara, CA) with specific primers listed in Supplementary Table 1 according to the manufacturer's protocol. DNA sequence and mutations were confirmed by Sanger sequencing. The wild-type and mutated *VCL* expression constructs were subsequently transfected into HeLa cells, the human cervical cancer cells (with expression of MYC-C), and subjected to immunofluorescent and pull-down assays.

Mice

Wnt1-Cre (strain# 022137), *Rosa26*^{YFP} (strain# 006148) and *Vcl*$^{f/f}$ (strain# 028451) mice were purchased from Jackson Lab. *Ptn* heterozygous knockout (T028403) mice were purchased from GemPharmatech. Mice were maintained in a mix outbred background of C57. Embryos were collected at different embryonic days dated from the day of the vaginal plug (E0.5). For each experiment, a representative result and the corresponding mean value ± SEM were

presented from at least five analyzed embryos. The number of individual embryos analyzed (n) is shown in all charts. Source data are provided as a Source Data file. Primers for genotyping are listed in Supplementary Table 2. Animals were kept in the Animal Laboratory of the University of Hong Kong, and all experiments were performed in accordance with procedures approved by the committee on the Use of Live Animals, the University of Hong Kong (CULATR 23-493 and CULATR 23-029). Our study examined male and female animals, and similar findings are reported for both sexes.

Generation of an inducible Cas9 human induced pluripotent stem cells (iCas9-hiPSCs)

A control hiPSC cell line (UE02302) was kindly provided by Dr. Guangjin Pan (Guangzhou Institutes of Biomedicine and Health, Chinese Academy of Sciences, China)(5). hiPSCs used in this study were at the intermediate (35-65) passage numbers and maintained on Matrigel (Corning, 354277)-coated plate with mTeSR1 medium (StemCell Technologies, 85850). To generate a hiPCS line with an inducible expression of Cas9, we adopted the doxycycline inducible Cas9 system published by Gonzalez *et al*(6). The inducible cassettes, one containing the iCas9 coding sequence under the regulation of the tight TRE promoter and the other carrying the M2rtTA tetracycline response element(7), were obtained from Addgene (access number #58409 and #60843). These two cassettes were sequentially targeted into the AAVS1 loci by CRISPR-Cas9D10a nickase-mediated homologous recombination with two gRNAs (Supplementary Table 1). It was followed by selection with puromycin and neomycin, and the properly targeted neomycin and puromycin resistant clones were selected using PCR. The inducible expression of Cas9 was validated by Western blot.

Derivation of enteric neural crest (ENCC) from iCas9- iPSC lines

At day 0, iCas9-hiPSCs were seeded on Matrigel-coated plate (10^5 cells cm^{-2}) in iPS cell medium containing 10 ng ml^{-1} FGF2 and 10 μM Y-27632 (Tocris Bioscience, 1254). Differentiation was then initiated by replacing iPSC medium with KSR medium, containing KnockOut DMEM (Thermo Fisher Scientific, 10829018) plus 15% KSR, 1% NEAA, 1% L-glutamine, 1X β-mercaptoethanol, 1% penicillin-streptomycin, LDN193189 (100 nM, Reprocell, 04-0074) and SB431542 (10 μM, Abcam, ab120163). The dual SMAD inhibitors and a potent GSK inhibitor were added at different time frame during the NC induction, including LDN193189 (from day 0 to day 3), SB431542 (from day 0 to day 4), 3 μM CHIR99021 (from day 2 to day 10, Reprocell, 04-0004). The NC cells were finally caudalized with 1 μM retinoic acid (Abcam, ab120728) (from day 6 to day 9). The KSR medium was gradually changed to N2 medium at day 4 by increasing N2 from 25% to 75% from day 4 to 9[8]. The N2 medium contained neural basal medium (Thermo Fisher Scientific, 22103049) and DMEM/F12 in 1:1 ratio supplemented with 0.5% N2 supplement (Thermo Fisher Scientific, 17502048), 1% B27 supplement (Thermo Fisher Scientific, 17504044), 5 μg ml^{-1} insulin (Thermo Fisher Scientific, 12585014) and 1% penicillin-streptomycin. The ENCCs were enriched by FACS with antibodies against $p75^{NTR}$ and HNK-1 at day 10 of the differentiation as described(8, 9).

In vitro differentiation of ENCCs to neuronal progenitors (NPs)

Around 40 thousand FACS-enriched ENCCs were seeded as droplets on poly-ornithine/laminin/fibronectin (PO/LM/FN)-coated 24 well plate in N2 medium containing 10ng ml^{-1} FGF2, 3 μM CHIR99021 and 10 μM Y-27632. The neuronal differentiation started 48 hours later and the attached ENCCs were then cultured with N2 medium containing BDNF (10ng ml^{-1} Peprotech, 450-01), GDNF (10 ng ml^{-1}, Peprotech, 450-10) and ascorbic acid (200

μM, Sigma, A4034-100G), NT-3 (10ng ml^{-1}, Peprotech, 450-03), NGF (10ng ml^{-1}, PeproTech, 450-01) and cAMP (1 μM, Sigma, D0260).

Fluorescence activated cell sorting (FACS)

The 10 day-differentiated cells were dissociated with Accutase and then incubated with antibodies against neural crest markers including APC-HNK-1, FITC-$p75^{NTR}$ and ITGA4-PE for 30-45 minutes on ice. The stained cells were washed and resuspended in PBS with 2% FBS. The HNK-1 and $p75^{NTR}$ double-positive cells were enriched with BD FACSAria III Cell Sorter (Becton Dickinson Immunocytometry Systems, San Jose, CA, USA). The HNK-1 and $p75^{NTR}$ double-positive cells were gated and sorted using the four-way purity mode and the purity of sorted cells was >96% and evaluated by flow cytometry. The sorted neural crest cells were collected for immunostaining or subsequent experiments. A list of primary antibodies and the working dilutions is provided in Supplementary Table 1. Isotype-matched antibodies were used as controls. FlowJo version 8.2 (Tree Star, Inc.)

Doxycycline-induced Cas9-mediated cleavage of VCL in ENCCs

Expression of Cas9 was induced on day 10, right after the FACS-enrichment or day 13 by addition of 2 μg ml^{-1} doxycycline (DOX), and the ENCCs were then transfected with sgRNA on day 12 or 15. On the day of transfection, the medium was replaced with neuronal differentiation medium without antibiotics. Predesigned Alt-R™ CRISPR-Cas9 sgRNAs targeting exon 1 (Hs.Cas9.VCL.1.AB) and exon 3 (Hs.Cas9.VCL.1.AA) of human *VCL* gene (Supplementary Table 1) were purchased from Integrated DNA Technologies (IDT, Coralville, USA). The sgRNAs were diluted in Opti-MEM and then mixed with Lipofectamine™ RNAiMAX (Invitrogen, USA) according to the manufacturer's protocol. The final concentration of each sgRNA used for transfection was 10 nM. The transfected cells were

collected at day 20 and day 30 during the neuronal lineage differentiation for immunofluorescence staining and Western blotting analyses.

Co-IP

co-IP was performed as previously described(10). Briefly, the human cells were lysed in 50 mM Tris-HCl, pH7.5, 100 mM NaCl, 1% Triton X-100, 0.1 mM EDTA, 0.5 mM $MgCl_2$, 10% glycerol, protease inhibitor cocktail (Roche), phosphatase inhibitor cocktail (Roche), and 10 μM pervanadate (NEB). Lysates were incubated with anti-GFP or anti-FLAG M2 Magnetic Beads (Sigma, M8823) overnight at 4 °C. Antibody/protein complexes were washed with lysis buffer for four times and analyzed by Western Blotting.

Immunoblots

hiPSC-derived ENCCs or embryonic guts were collected and then lysed with cell lysis buffer containing 20 mM Tris (pH 7.5), 150 mM NaCl, 1 mM EDTA, 1 mM EGTA, 1% Triton X-100, 2.5 mM sodium pyrophosphate, 1 mM β-glycerophosphate, 1 mM Na_3VO_4, 1 μg/mL leupeptin and 1 mM phenylmethanesulfonyl fluoride (PMSF). Cell lysates containing 5-25 μg of total protein were separated on 8-12% SDS-polyacrylamide gels and blotted onto nitrocellulose membranes. The membranes were then incubated with primary antibodies (Supplementary Table 3). The same membranes were probed with a 1:5000 dilution of anti-actin monoclonal or anti-Gapdh antibody to ensure equal loading of cell protein per lane. All blots were incubated with 1:5000 dilutions of secondary horseradish peroxidase-conjugated anti-mouse or anti-rabbit antibody (Supplementary Table 4). Antibody-bound proteins were visualized using a chemiluminescence system (Amersham Pharmacia Biotech). The representative pictures of at least 3 independent assays were shown.

Acetylcholinesterase (AChE) stain

Embryonic guts at E18.5 were first dissected in PBS and fixed in 4% PFA for 2-4 hours at 4ºC. The Acetylcholinesterase Rapid Staining Kit (MBL #8450) was used to stain the ENS following the manufacturer's protocol. The whole-mount images were captured using Leica MZ10F stereomicroscope.

Immunofluorescence studies

Immunocytochemistry

Immunocytochemistry - Cells were fixed with 4% PFA in PBS at room temperature for 30 min, followed by blocking with 1% bovine serum albumin (BSA) (Thermo Scientific, 23209) with 0.1% Triton X-100 (Sigma, T8787) in PBS. Cells were then incubated in primary antibody overnight at 4°C, followed by host-appropriate Alexa Fluor -488 or 594 secondary antibody (Molecular Probes, Invitrogen) (Tables S3 & 4) for 1 h at room temperature. Cells were then counterstained with 1:2000 DAPI (Thermo Fisher Scientific, 62248) in PBS for 10 min at room temperature to detect nuclei and mounted with ProLong Diamond Antifade Mountant with DAPI (Thermo Fisher Scientific, P36971). All the fluorescence images were acquired by Carl Zeiss LSM800 or LSM900 confocal microscope. Quantitative image analysis of differentiated cells was performed with ImageJ plug-in tool. The total signal of the specific marker was normalized with DAPI signal and the values reported in bar charts represent the mean ± SEM.

Whole mount immunohistochemistry - Embryonic guts were first dissected in PBS, fixed in 4% PFA for 2-4 hours at 4ºC. The dissected guts were then washed with 1% PBST for 30 minutes at RT for 3 times and incubated in blocking buffer (1% PBST with 10% FBS and 0.2% sodium azide) for 2 hours at RT. Subsequently, they were incubated with primary antibodies (diluted in PBS with 20% DMSO and 5% donkey serum) at 4ºC for 2-3 days. The guts were

washed with 1% PBST for 10 minutes for 3 times and incubated with secondary antibody for 2-3 days at RT. After washing with 1% PBST for 10 minutes for 3 times. The guts were dehydrated in 24-well dishes in graded methanol/ PBS (approximately 500 mL/well, extra if needed to cover, 30 minutes/wash: 50% methanol, 70% methanol, 80% methanol, 95% methanol, 100% methanol x3, room temperature, on rocker). The rehydrated guts were then incubated in Murray's Clear (2:1 benzyl benzoate: benzyl alcohol) until completely translucent (15–30 minutes, room temperature). Guts were then mounted on glass slides in Murray's Clear and imaged within 48 hours as described previously[11]. Whole mount immunofluorescence images were captured using Carl Zeiss LSM 800 or LSM900 confocal microscope.

Immunohistochemistry - For section immunohistochemistry, embryos were fixed in 4% paraformaldehyde (PFA) in PBS at 4ºC, dehydrated and cryoprotected in 30% sucrose in PBS at 4ºC and embedded in OCT compound (Tissue-Tek). The sections were blocked in PBS containing 10% normal goat or horse serum (DAKO) for 1 hour at room temperature, then incubated overnight at 4ºC in a mixture of the primary antibodies (Supplementary Table 3). After washing, the immunosignals were then detected using the secondary antibody conjugated with Alexa Fluor 488 or 594 (Supplementary Table 4). Tissue sections were photographed using a Carl Zeiss LSM 800 or LSM900 confocal microscope.

Confocal microscopy and live imaging - while section immunofluorescence images were captured using. Carl Zeiss LSM 800 was also used for live imaging where Z-stack images were captured every 5 minutes for 12-15 hours. Live imaging was performed on E12.5 guts using the conditions described previously(11, 12). Immunofluorescence images were processed and analysed using ImageJ (NIH). For live imaging of ENCC migration, ENCCs were tracked by Manual Tracking plugin and the tracks generated were analysed using Chemotaxis and

Migration Tool plugin (ibidi) to calculate cell speed, net speed and persistence. Persistence of ENCC migration was calculated as the ratio of net distance travelled by ENCC to the total distance travelled by ENCC.

Focal adhesion size analysis

Images were processed using ImageJ version 2.16. For calculating focal adhesion size, cells were stained with Paxillin or VCL. The captured FITC or Texas-Red images were background-subtracted, and a threshold was set to restrict analysis to FA. The images were then converted to binary mode, and the FA size (area) per cell was measured using ImageJ plug-in software. The average FA size of 60 cells from three independent assays is shown in the bar chart (mean ± SEM).

Droplet-based single-cell RNA-sequencing (scRNA-seq)

The FACS-sorted cells were then subjected to droplet-based scRNA-seq using Chromium Single Cell platform and Single Cell 3' Library Kits (10x Genomics) in Centre of PanorOmic Science (CPOS), The University of Hong Kong. In brief, cells were encapsulated into Gel Beads-in-emulsion (GEMs) by the 10X Chromium Single Cell Controller, followed by reverse transcription and library preparation to become a pool of cDNA libraries. Libraries were then purified and sequenced on an Illumina^{TM} NextSeq 500 according to the manufacturer's protocol.

Processing and quality control of single-cell RNA sequencing data - To address potential variations introduced by different software versions, we reprocessed our previously published scRNA-seq data of YFP-labelled ENCCs at E13.5(11). FASTQ files of control and *Vcl* mutant samples at E13.5 and E15.5 were processed and aligned to the mm10 mouse reference

transcriptome (mm10-2020-A-2.0.0) to generate gene-by-cell matrices, using the 10x Genomics Cellranger software (v7.2.0) with default parameters. The gene-by-cell matrices were loaded in the Seurat(13) package for the quality control. To mitigate the doublet, we filtered the cells on nCount_RNA metrics. For E13.5 samples, cells were filtered based on the criteria “500 < nFeature_RNA & nFeature_RNA < 7500 & percent.mt < 5 & nCount_RNA < 40000”. For E15.5 samples, cells were retained if “percent.mt < 5 & nCount_RNA < 2e+05 & nFeature_RNA > 200”. Finally, we got 30,157 cells as shown in Supplementary Data 2.

Single-cell RNA-seq data integration and annotation - Control data at E13.5 and E15.5 were integrated for main cell type annotation. Expression matrices were normalized using the “LogNormalize” method in Seurat. After scaling the data, principal component analysis (PCA) was performed using the “RunPCA” function on the top 2,000 variable genes for each of the four sample datasets separately. The control data from both stages were then integrated using the anchor-based reciprocal PCA integration method implemented in the Seurat R package. Cells were finally annotated as bipotent progenitors (BP), glial progenitors (GP), neurons, and enteric mesothelial fibroblasts (ENMFB) based on the expression of canonical marker genes and proliferative markers as shown in Figure 5C. Using annotations in control data as a reference, *Vcl* mutant data were annotated via the label transfer method in Seurat and based on the clustering results.

To identify the branching neuronal lineage, data in this study were integrated with the scRNA-seq data of the enteric nervous system of the small intestine at E18.5(14). The E18.5 data were reprocessed and annotated as GP, BP, Neuroblast, Branch A and Branch B neurons based on the canonical marker gene expression and branch-specific transcriptional factors identified in the original study(14). After integrating the data from all three stages using RPCA anchor-

based method, the neuron cells at E13.5 and E15.5 were categorized as Neuroblast, Branch A or Branch B based on the label transfer results and clustering results in the integrated UMAP embedding. Marker genes for each cell type were identified for validation, using the "FindAllMarkers" function in Seurat, with the parameter "min.pct" set to 0.25. The marker information is shown in Supplementary Data 3.

Proportion analysis

Due to sample size constraints, the direct statistical testing on the comparison of cell type proportion was not feasible. To address this, we employed scanpro(15) (a tool designed for proportion analysis in single-cell sequencing data) to perform the proportion analysis for statistical evaluation. In our case, scanpro generated pseudo-replicates on each sample and used them for the following Student's *t*-test with the transform model setting as "arcsin".

RNA velocity analysis - The RNA velocity is inferred independently for the control and *Vcl* mutant datasets at E13.5 and E15.5 data using velocyto v0.17 (http://velocyto.org/velocyto.py/index.html), The results were subsequently integrated for comprehensive velocity visualisation using scVelo(16).

Pseudotime inference - To remove the cell number effect, we standardized the total number of cells (3404) in disrupted cell states in control and mutant groups for the pseudotime inference. As the distribution of cells in principal components 1 (PC1) and 3 (PC3) reflected a continuous pseudotemporal trajectory from BP-Branch A lineage, the pseudotime was inferred using SlingShot(17) based on these two dimensions. In this analysis, BP is designated as the starting cluster, with Branch A as the terminal cluster.

Differential expression analysis and pathway enrichment analysis - Monocle v2.30.1(18) was used to identify the differentially expressed genes between control and mutant in the corresponding cell states. Genes with FDR-adjusted *P* value less than 0.05 were considered significant and used in downstream analysis. UP and down-regulated genes are determined by the mean raw count of each gene in the control and mutant cells. Gene ontology (GO) term enrichment analyses and Kyoto Encyclopedia of Genes and Genomes (KEGG) enrichment analyses were performed using clusterProfiler(19) R package. Terms that had an adjusted *P* value < 0.01 were defined as significantly enriched.

Gene regulatory network inference - The DEGs of E13.5 BP, E15.5 Neuroblast, E13.5 Branch A and E15.5 Branch A were used for GO enrichment analysis. Among the top 50 pathways ranked by the number of DEGs, the 10 most disrupted pathways in each cell state were identified by the log2 fold changes of the pathway scores between control and *Vcl* mutants. These scores were calculated using all the genes involved in the pathways by AUCell(20). Next, we grouped the DEGs from the 10 most disrupted pathways in each cell state using the gene regulatory network inference strategy implemented in SCENIC[19] R package. This analysis utilized the mouse TFs reference from CisTarget (https://resources.aertslab.org/cistarget/) and the motif scores within the 10 kb flanking regions of the TSS of the genes (https://resources.aertslab.org/cistarget/databases/mus_musculus/mm10/refseq_r80/mc9nr/gene_based/). Of 640 DEGs, 447 genes were identified as the target genes of *E2f1*, *Egr1*, and *Klf7*. Then, these target genes were categorized as four modules based on the regulatory relationship. Based on TF expression and regulon activity along the pseudotemporal trajectory, the modules were assigned to their corresponding cell states. GO enrichment was performed on the genes within modules, where the top 2 pathways ranked by the gene count were

presented. To assess the directionality of changes in the associated biological processes, module scores were calculated using the AUCell(20).

Protein-protein interaction (PPI) network analysis - The genes in modules and *Vcl* were uploaded to STRING database for the PPI inference. To ensure reliable interactions, we exclusively included PPIs derived from curated databases and experimental evidence, and integrated them into the gene regulatory network. Specifically, we selected only "Experiments" and "Databases" as active interaction sources on the STRING website, with the medium confidence score set to the default value of 0.4 in the "minimum required interaction score" section of the settings.

Spearman correlation - Spearman correlations were calculated using the “corr.test” function in R. Only the gene pairs with P value < 0.05 were kept. Genes detected but not included in the modules were designated as the background group. The comparisons of correlations used two-sided Wilcoxon test.

Spatial transcriptomic analysis

Spatial transcriptomics was carried out using the 10x Genomics Visium platform with Visium Spatial Gene Expression Slides & Reagent kits (PN-1000187). 16 μm sagittal sections from OCT-embedded fresh frozen control (*Wnt1-Cre*, *Rosa26*^{YFP}) and mutant (*Wnt1-Cre*, *Rosa26*^{YFP};*Vcl*$^{f/f}$) embryos at E13.5 were mounted onto the Visium Spatial slides. The innate YFP signals on the fresh-frozen sections were captured using Nikon Ti2-E Widefield Microscope for the subsequent identification of NCC derivatives in the sections. The sections were then fixed with ice-cold methanol for 20 min and counterstained with DAPI and Alexa Fluor 594 Phalloidin (Thermo Fisher Scientific, A12381) to detect nuclei. The images were

captured using the same Nikon Ti2-E Widefield fluorescent microscope and different fluorescence channels were aligned using Carl Zeiss ZEN lite software. The stained sections were then permeabilized for 12 min to release mRNAs, which bind to the spatially barcoded-oligos present in the underlying spots and reverse transcribed, according to the manufacturer's protocol. Libraries prepared from the cDNAs were sequenced on the Illumina™ NovaSeq 6000 at >50,000 reads per spot generating >400M reads per section. Spaceranger software (version 3.1.0, 10x Genomics) was used to align and obtain raw counts from each of the spots on the Visium spatial transcriptomics slides against the GRCm39 mouse genome reference data.

Processing of the spatial transcriptomic data - Sequencing read alignment, fiducial/tissue detection, and spot barcode/UMI counting of the spatial transcriptomic data were performed using the 10x Genomics toolkit Space Ranger (v2.0.1) with a modified reference genome (mm10-2020-A) with YFP gene contig added. Downstream analysis was conducted using Seurat. Quality control included removal of spots with fewer than 10K unique molecular identifiers (UMI) or fewer than 4K expressed genes.

Cell type assignment in spatial transcriptome data - Initially, the gut regions were manually selected based on the histological images using Loupe Browser 8 from 10x Genomics (https://www.10xgenomics.com/cn/products/loupe-browser). These regions were then further defined based on the expression of marker genes of gut regions. For the large intestine, small intestine, and stomach regions, spots were assigned based on the deconvolution results of CARD(21) using the corresponding scRNA-seq dataset of embryonic guts from the published dataset(22). Employing a similar strategy, the spots were further annotated as epithelial (EPI), mesenchymal cells (MES), and neural crest cells (NC). Specifically, the spots with scaled *YFP* expression higher than 1 were annotated as NC cells.

Cell-cell interactions inference - The cell-cell interactions were inferred using Cellchat v1.61(23). Specifically, we focused on the DEGs when inferring the cell-cell interactions on scRNA-seq data. Interactions that included at least one DEG in both the ligand and receptor were retained. Those with a *P* value < 0.05 were considered significant. The loss and gain interactions in *Vcl* mutant were finally visualized using dot plot. In the spatial transcriptome data, we focused on the large intestine regions that share similar spatial locations, because the most pronounced aberrant phenotype was observed in the colon of mice. Few EPI were identified in the large intestine; thus, the EPI in other regions were also included in the communication inference. Given that disrupted interactions predominantly involve NC and EPI, and the *Vcl* deletion is effective only in NC, our analysis concentrated on interactions within NC and other cell types.

The Major Resources Tables are provided as the Supplemental Material.

References:

1. Gui H, et al. Whole exome sequencing coupled with unbiased functional analysis reveals new Hirschsprung disease genes. *Genome Biol.* 2017;18(1):48.
2. Tang CS, et al. Identification of Genes Associated With Hirschsprung Disease, Based on Whole-Genome Sequence Analysis, and Potential Effects on Enteric Nervous System Development. *Gastroenterology.* 2018;155(6):1908-22 e5.
3. Tang CS, et al. Uncovering the genetic lesions underlying the most severe form of Hirschsprung disease by whole-genome sequencing. *Eur J Hum Genet.* 2018;26(6):818-26.
4. Li MX, et al. A comprehensive framework for prioritizing variants in exome sequencing studies of Mendelian diseases. *Nucleic Acids Res.* 2012;40(7):e53.
5. Xue Y, et al. Generating a non-integrating human induced pluripotent stem cell bank from urine-derived cells. *PLoS One.* 2013;8(8):e70573.
6. Gonzalez F, et al. An iCRISPR platform for rapid, multiplexable, and inducible genome editing in human pluripotent stem cells. *Cell Stem Cell.* 2014;15(2):215-26.
7. DeKelver RC, et al. Functional genomics, proteomics, and regulatory DNA analysis in isogenic settings using zinc finger nuclease-driven transgenesis into a safe harbor locus in the human genome. *Genome Res.* 2010;20(8):1133-42.
8. Lai FP, et al. Correction of Hirschsprung-Associated Mutations in Human Induced Pluripotent Stem Cells Via Clustered Regularly Interspaced Short Palindromic Repeats/Cas9, Restores Neural Crest Cell Function. *Gastroenterology.* 2017;153(1):139-53 e8.
9. Lau ST, et al. Activation of Hedgehog Signaling Promotes Development of Mouse and Human Enteric Neural Crest Cells, Based on Single-Cell Transcriptome Analyses. *Gastroenterology.* 2019;157(6):1556-71 e5.
10. Li P, et al. alphaE-catenin inhibits a Src-YAP1 oncogenic module that couples tyrosine kinases and the effector of Hippo signaling pathway. *Genes Dev.* 2016;30(7):798-811.
11. Lai FP, et al. Ciliary protein Kif7 regulates Gli and Ezh2 for initiating the neuronal differentiation of enteric neural crest cells during development. *Sci Adv.* 2021;7(42):eabf7472.
12. Liu JA, et al. Identification of GLI Mutations in Patients With Hirschsprung Disease That Disrupt Enteric Nervous System Development in Mice. *Gastroenterology.* 2015;149(7):1837-48 e5.
13. Hao Y, et al. Dictionary learning for integrative, multimodal and scalable single-cell analysis. *Nat Biotechnol.* 2024;42(2):293-304.
14. Morarach K, et al. Diversification of molecularly defined myenteric neuron classes revealed by single-cell RNA sequencing. *Nat Neurosci.* 2021;24(1):34-46.
15. Alayoubi Y, et al. Scanpro is a tool for robust proportion analysis of single-cell resolution data. *Sci Rep.* 2024;14(1):15581.
16. Bergen V, et al. Generalizing RNA velocity to transient cell states through dynamical modeling. *Nat Biotechnol.* 2020;38(12):1408-14.
17. Street K, et al. Slingshot: cell lineage and pseudotime inference for single-cell transcriptomics. *BMC Genomics.* 2018;19(1):477.
18. Qiu X, et al. Reversed graph embedding resolves complex single-cell trajectories. *Nat Methods.* 2017;14(10):979-82.
19. Yu G, et al. clusterProfiler: an R package for comparing biological themes among gene clusters. *OMICS.* 2012;16(5):284-7.
20. Aibar S, et al. SCENIC: single-cell regulatory network inference and clustering. *Nat Methods.* 2017;14(11):1083-6.

21. Ma Y, et al. Spatially informed cell-type deconvolution for spatial transcriptomics. *Nat Biotechnol.* 2022;40(9):1349-59.
22. Zhao L, et al. Mesenchymal-epithelial interaction regulates gastrointestinal tract development in mouse embryos. *Cell Rep.* 2022;40(2):111053.
23. Jin S, et al. Inference and analysis of cell-cell communication using CellChat. *Nat Commun.* 2021;12(1):1088.
