# Supplementary figures and images for: Vinculin influences essential processes in enteric nervous system development and Hirschsprung disease pathogenesis
Source: J Clin Invest. 2025 Dec 9;136(3):e198531. doi: 10.1172/JCI198531 (PMC12867144; doi:10.1172/JCI198531)

Fig 1E

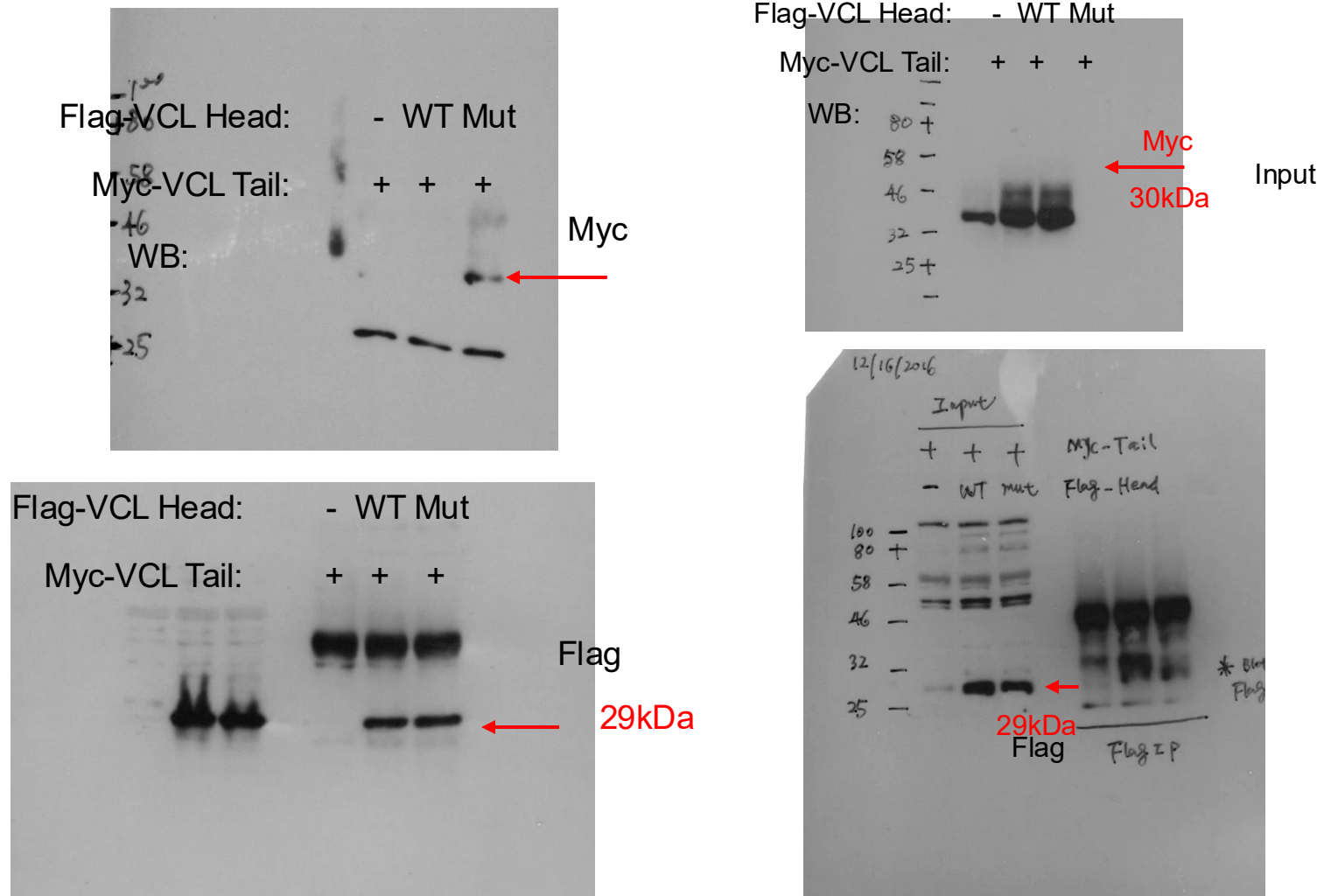



Fig 5J

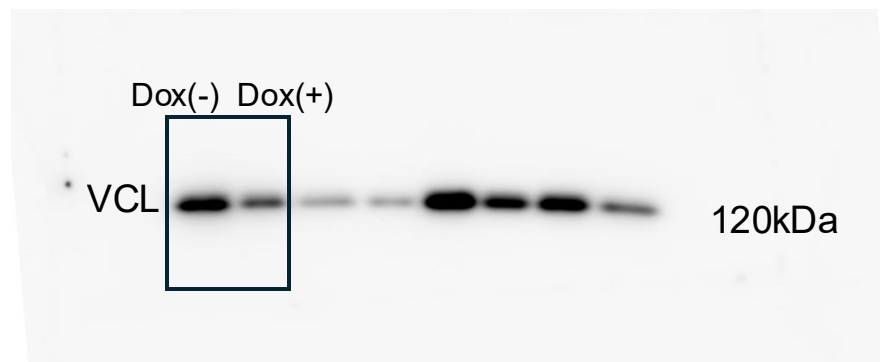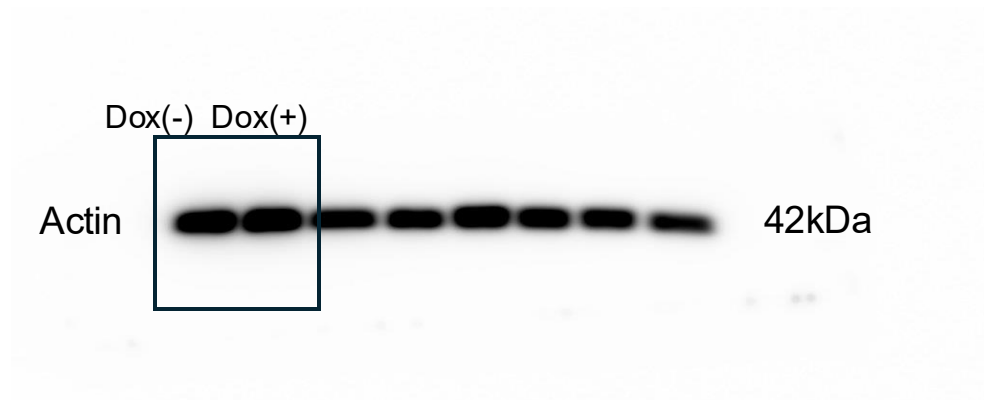

KD at Day12

hNP<sub>early</sub>D20

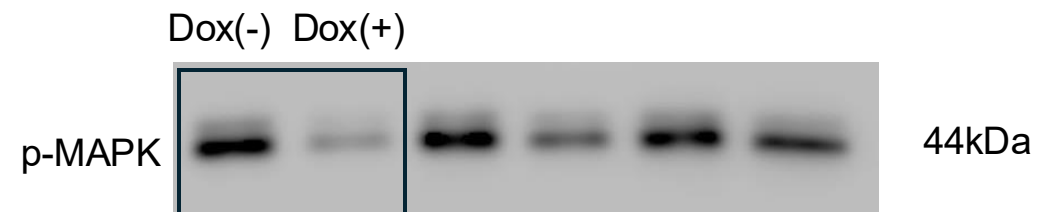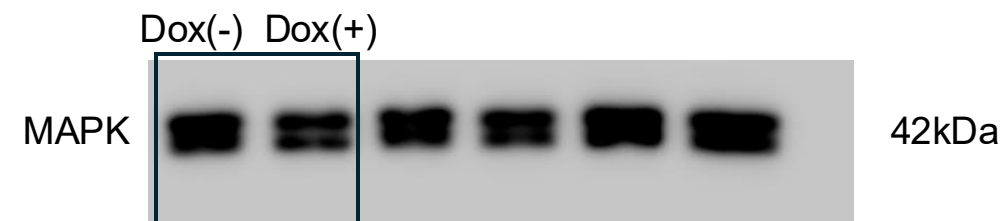

Fig 6G

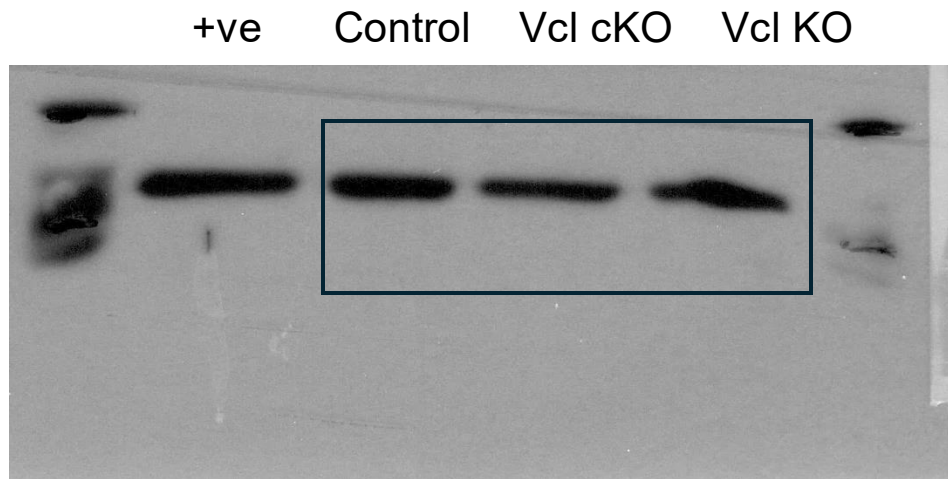

35kDa  
**Gapdh**

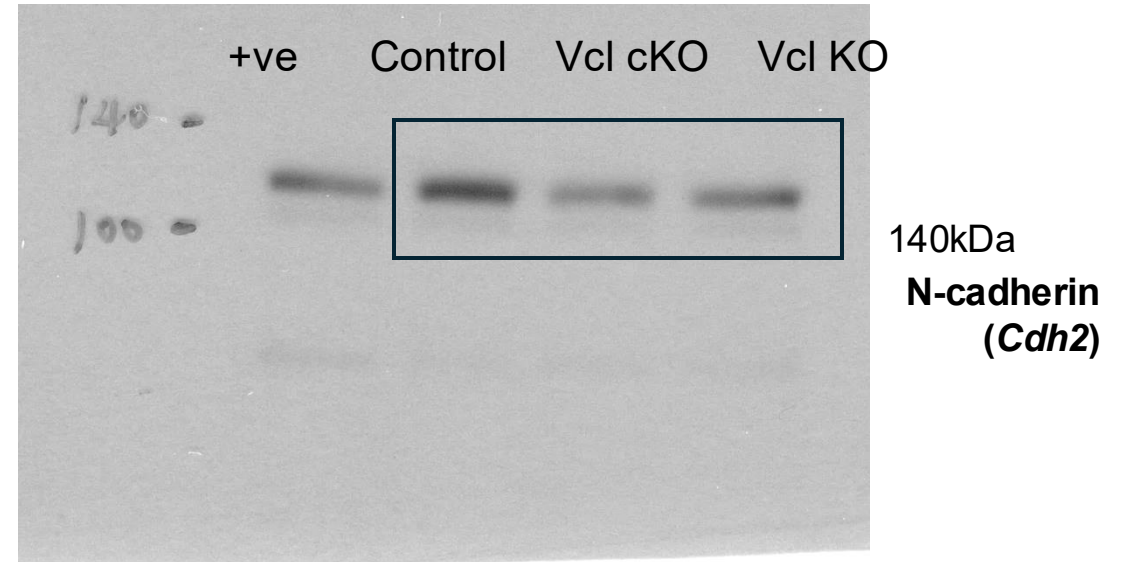

140kDa  
**N-cadherin (Cdh2)**

Fig S9

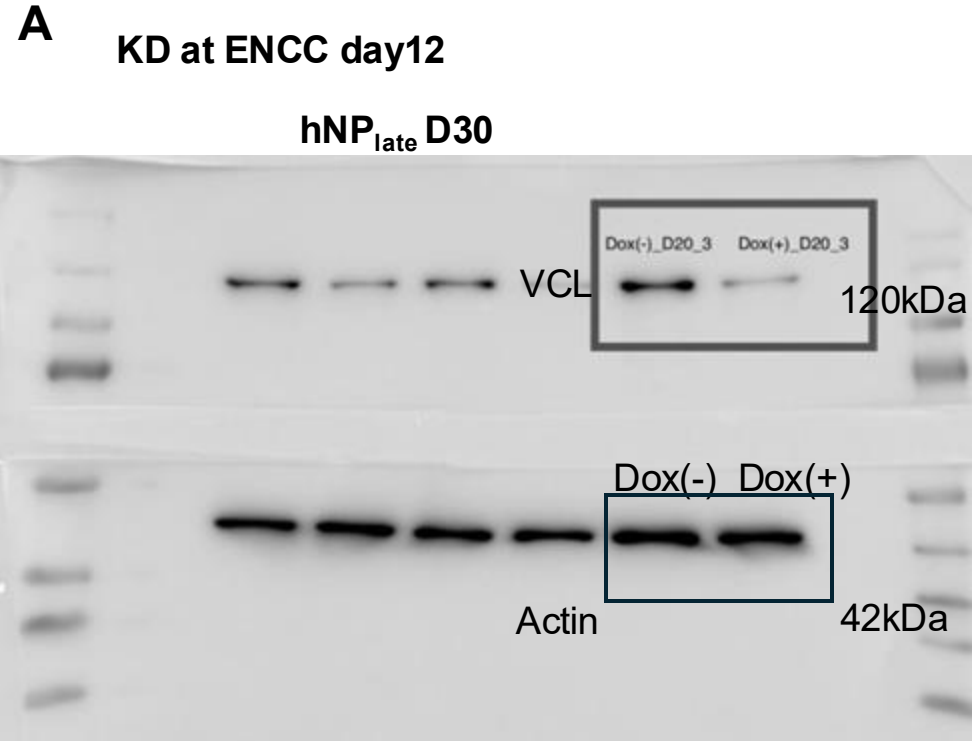

**B**

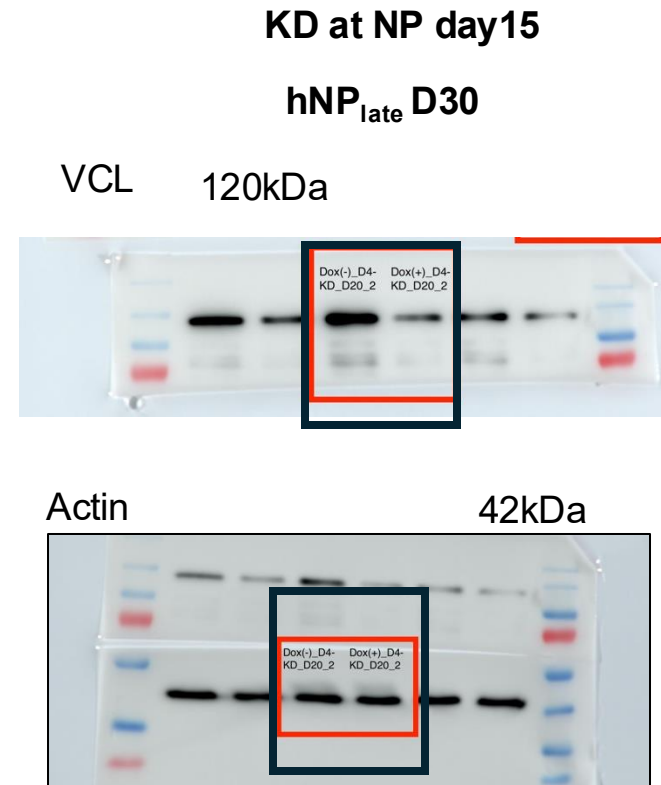

Supplement: Unedited blot and gel images [file jci-136-198531-s116.pdf]
